# Supplementary material for: Association of analgosedation with psychiatric symptoms and health-related quality of life in ARDS survivors: Post hoc analyses of the DACAPO study
Source: PLoS One. 2022 Oct 21;17(10):e0275743. doi: 10.1371/journal.pone.0275743 (PMC9586389; doi:10.1371/journal.pone.0275743)
Supplement: S1 Table — *p <0.05; **per 1000 mg; #further adjusted for age, sex, SAPS, days on ICU; B: regression coefficient, 95%-CI: 95% confidence interval, PHQ-9: Patient Health Questionnaire-9; PTSS-14: Post-Traumatic Stress Syndrome 14-Questions Inventory; asymptoms of psychiatric disorders (depression and PTSD) were diagnosed according to the results of patient self-reported questionnaires. (PDF) [file pone.0275743.s001.pdf]

**S1 Table. Multiple linear regression models of the influence of cumulative analgosedation (without ketamine and midazolam) on psychiatric symptoms in ARDS survivors 3, 6 and 12 months after ICU discharge.** S1 Table legend: *p <0.05; **per 1000 mg; #further adjusted for age, sex, SAPS, days on ICU; B: regression coefficient, 95%-CI: 95% confidence interval, PHQ-9: Patient Health Questionnaire-9; PTSS-14: Post-Traumatic Stress Syndrome 14-Questions Inventory; asymptoms of psychiatric disorders (depression and PTSD) were diagnosed according to the results of patient self-reported questionnaires

| PHQ-9 - 3 months after ICU discharge [a,#] | B | 95%-CI | | p-value |
|---|---|---|---|---|
| Propofol applied | 6,895 | 0,072 | 13,718 | 0,058 |
| Cumulative propofol dose ** | 0,000 | 0,000 | 0,000 | 0,782 |
| Esketamin applied | -9,836 | -21,479 | 1,806 | 0,096 |
| Cumulative esketamine dose ** | 0,001 | -0,001 | 0,002 | 0,469 |
| Clonidine applied | -0,871 | -3,767 | 2,024 | 0,550 |
| Cumulative clonidine dose ** | 0,054 | -0,019 | 0,126 | 0,147 |
| Dexmedotomidine applied | 1,919 | -1,859 | 5,697 | 0,314 |
| Cumulative dexmedetomidine dose ** | 0,000 | -0,001 | 0,000 | 0,703 |
| Sufentanil applied | -6,998 | -12,371 | -1,625 | 0,012 |
| Cumulative sufentanil dose ** | 0,000 | 0,000 | 0,000 | 0,705 |

| PHQ-9 - 6 months after ICU discharge [a,#] | B | 95%-CI | | p-value |
|---|---|---|---|---|
| Propofol applied | 5,145 | -1,952 | 12,243 | 0,152 |
| Cumulative propofol dose ** | 0,000 | 0,000 | 0,000 | 0,747 |
| Esketamin applied | -4,696 | -18,288 | 8,896 | 0,492 |
| Cumulative esketamine dose ** | 0,000 | -0,002 | 0,001 | 0,400 |
| Clonidine applied | 0,184 | -2,992 | 3,360 | 0,908 |
| Cumulative clonidine dose ** | 0,047 | -0,028 | 0,122 | 0,218 |
| Dexmedotomidine applied | 0,371 | -3,805 | 4,547 | 0,860 |
| Cumulative dexmedetomidine dose ** | 0,000 | 0,000 | 0,001 | 0,473 |
| Sufentanil applied | -3,201 | -9,166 | 2,764 | 0,287 |
| Cumulative sufentanil dose ** | 0,000 | 0,000 | 0,000 | 0,734 |

| PHQ-9 - 12 months after ICU discharge [a,#] | B | 95%-CI | | p-value |
|---|---|---|---|---|
| Propofol applied | 2,764 | -4,604 | 10,131 | 0,456 |
| Cumulative propofol dose ** | 0,000 | 0,000 | 0,000 | 0,746 |
| Esketamin applied | -4,686 | -18,188 | 8,876 | 0,493 |
| Cumulative esketamine dose ** | 0,000 | -0,002 | 0,002 | 0,806 |
| Clonidine applied | -2,351 | -5,679 | 0,977 | 0,163 |
| Cumulative clonidine dose ** | 0,024 | -0,057 | 0,106 | 0,550 |
| Dexmedotomidine applied | -0,198 | -5,147 | 4,752 | 0,937 |
| Cumulative dexmedetomidine dose ** | 0,000 | -0,001 | 0,001 | 0,637 |
| Sufentanil applied | -3,066 | -8,837 | 2,705 | 0,292 |
| Cumulative sufentanil dose ** | 0,000 | 0,000 | 0,000 | 0,674 |

| PTSS-14 - 3 months after ICU discharge [a,#] | B | 95%-CI | | p-value |
|---|---|---|---|---|
| Propofol applied | 24,201 | 3,787 | 44,615 | 0,061 |
| Cumulative propofol dose ** | 0,000 | 0,000 | 0,000 | 0,069 |
| Esketamin applied | -21,582 | -55,556 | 12,392 | 0,209 |
| Cumulative esketamine dose ** | 0,001 | -0,003 | 0,005 | 0,636 |
| Clonidine applied | 1,088 | -6,901 | 9,077 | 0,786 |
| Cumulative clonidine dose ** | 0,159 | -0,050 | 0,368 | 0,133 |
| Dexmedotomidine applied | -2,873 | -13,572 | 7,826 | 0,593 |
| Cumulative dexmedetomidine dose ** | -0,001 | -0,003 | 0,001 | 0,274 |
| Sufentanil applied | -13,874 | -31,076 | 3,327 | 0,112 |
| Cumulative sufentanil dose ** | 0,000 | 0,000 | 0,001 | 0,400 |

| PTSS-14 - 6 months after ICU discharge [a,#] | B | 95%-CI | | p-value |
|---|---|---|---|---|
| Propofol applied | 13,982 | -13,079 | 41,043 | 0,305 |
| Cumulative propofol dose ** | 0,000 | 0,000 | 0,000 | 0,693 |
| Esketamin applied | -19,345 | -64,863 | 26,173 | 0,399 |
| Cumulative esketamine dose ** | -0,002 | -0,006 | 0,002 | 0,351 |
| Clonidine applied | 1,919 | -10,068 | 13,905 | 0,750 |
| Cumulative clonidine dose ** | 0,200 | -0,069 | 0,470 | 0,143 |
| Dexmedotomidine applied | -6,965 | -22,615 | 8,684 | 0,377 |
| Cumulative dexmedetomidine dose ** | 0,001 | -0,001 | 0,004 | 0,207 |
| Sufentanil applied | -3,121 | -24,580 | 18,338 | 0,772 |
| Cumulative sufentanil dose ** | 0,000 | -0,001 | 0,000 | 0,695 |

| PTSS-14 - 12 months after ICU discharge [a,#] | B | 95%-CI | | p-value |
|---|---|---|---|---|
| Propofol applied | 7,815 | -17,659 | 33,289 | 0,542 |
| Cumulative propofol dose ** | 0,000 | 0,000 | 0,000 | 0,554 |
| Esketamin applied | -19,348 | -64,963 | 26,373 | 0,389 |
| Cumulative esketamine dose ** | 0,001 | -0,005 | 0,007 | 0,821 |
| Clonidine applied | 3,071 | -7,966 | 14,108 | 0,580 |
| Cumulative clonidine dose ** | 0,041 | -0,223 | 0,306 | 0,756 |
| Dexmedotomidine applied | -3,078 | -18,327 | 12,171 | 0,688 |
| Cumulative dexmedetomidine dose ** | 0,000 | -0,002 | 0,003 | 0,847 |
| Sufentanil applied | -5,542 | -26,425 | 15,341 | 0,597 |
| Cumulative sufentanil dose ** | 0,000 | 0,000 | 0,001 | 0,441 |
