# Supplementary material for: Association of analgosedation with psychiatric symptoms and health-related quality of life in ARDS survivors: Post hoc analyses of the DACAPO study
Source: PLoS One. 2022 Oct 21;17(10):e0275743. doi: 10.1371/journal.pone.0275743 (PMC9586389; doi:10.1371/journal.pone.0275743)
Supplement: Attachment — Submitted filename: Response to Reviewers 090822.docx [file pone.0275743.s003.docx]

**Response letter**

Dear Mrs. Linda L. Maerz,

Thank you for the opportunity to revise our manuscript entitled: “Association of analgosedation with psychiatric symptoms and health-related quality of life in ARDS survivors: Post hoc analyses of the DACAPO study”. We are very grateful for the positive review, helpful suggestions, and comments of the reviewers and you to improve our paper. We have thoroughly modified the manuscript accordingly. Detailed responses to the reviewers’ comments are listed below.

**Journal Requirements:**

When submitting your revision, we need you to address these additional requirements.

1. Comment (C): Please ensure that your manuscript meets PLOS ONE's style requirements, including those for file naming.

Answer (A): We modified the whole manuscript including the title page and file naming.

2. C: We noticed you have some minor occurrence of overlapping text with a previous publication.

A: We rephrase parts of the methods section and cited our previous publication (page 4 to 5).

3. C: In your Data Availability statement, you have not specified where the minimal data set underlying the results described in your manuscript can be found. PLOS defines a study's minimal data set as the underlying data used to reach the conclusions drawn in the manuscript and any additional data required to replicate the reported study findings in their entirety. All PLOS journals require that the minimal data set be made fully available. For more information about our data policy, please see <http://journals.plos.org/plosone/s/data-availability>. Important: If there are ethical or legal restrictions to sharing your data publicly, please explain these restrictions in detail. Please see our guidelines for more information on what we consider unacceptable restrictions to publicly sharing data: http://journals.plos.org/plosone/s/data-availability#loc-unacceptable-data-access-restrictions. Note that it is not acceptable for the authors to be the sole named individuals responsible for ensuring data access.

A: The conceptual design of the informed consent did not allow sharing of the results of the self-report questionnaires. We described it in more detail in the Declarations section (page 15).

“The study data cannot be shared publicly because the written consent obtained from the study participants did not include a public availability of their data. Data are available from the two principal investigators (PIs) Christian Apfelbacher and Thomas Bein (E-mail: [Christian.Apfelbacher@med.ovgu.de](mailto:Christian.Apfelbacher@med.ovgu.de) and/or [Thomas.Bein@ukr.de](mailto:Thomas.Bein@ukr.de)) on request.”

4. C: Address all items indicated by tracked changes in the attached Word document (PONE-D-22-09940 [ed])

A: All questions which were addressed could hopefully be adequately answered. Please look at the revised manuscript with tracked changes.

**Reviewer #1:**

1. Comment (C): Perhaps the main concern is the interaction of different sedatives/analgesics used in the management of ARDS patients included in this evaluation. It is common to switch between therapies or administer concomitant sedatives/analgesics to achieve the appropriate level of sedation. It was not clear if these interactions were accounted for. The study results indicate that ketamine was associated with an increased risk of psychiatric symptoms. However, it's not clear if any administration of ketamine was associated with these findings or if a certain cohort of ketamine patients (those who received other sedatives or analgesics) had an increased risk of psychiatric symptoms. For example, patients who received ketamine and midazolam might have had an increased risk of psychiatric symptoms versus patients who received ketamine and propofol. Furthermore, it would be helpful if authors report in detail the breakdown of sedatives and analgesics (number of patients who received one sedative, two sedatives, and three sedatives, etc.)

A: Thank you for your critical note. Our data do not allow us to say, which drugs were switched against each other or which were stopped first. We added the multiple drug application to the multiple linear regression analyses but found no influence on the existing results. As stated below we hope we could explain our interesting findings in more detail (page 8 and 10).

“In the following, the number of sedative and analgesic agents that patients received during ICU treatment are listed (based on Table 2): 12 patients (8.9%) got a sixfold, 28 patients (20.9%) fivefold, 38 patients (28.4%) fourfold, 34 patients (25.4%) a threefold, 19 patients (14.2%) twofold and only 3 patients (2.2%) single analgosedation.”

“Thirty-two of 39 patients (82.1%) with ketamine application and all patients with esketamine additionally received midazolam.”

“The number of drugs for analgosedation showed no influence on the psychiatric outcome in the multivariable linear regression analysis.”

2. C: The other concern in the design of this evaluation is variation in practice. The study utilized the DACAPO database. Different institutions have various sedatives and analgesic dosing regimens, titration protocols goals, and ARDS management guidelines. It would be helpful to account in the multivariable analysis for the type of institutions and variation in practice.

A: We were very thankful to the reviewer for this important note. We addressed this fact in more detail in the methods section (page 5 and 6).

“The type of analgesia and sedation, duration and cumulative doses of drugs were extracted and transmitted from the patient data management system of nine recruiting study centres (8 University hospitals and one hospital for basic and standard care).”

3. C: It was not clear from the study's design whether all sedatives and analgesics extracted from the database were accounted for. According to Table 2, none of the patients received commonly used sedatives and analgesics such as dexmedetomidine, fentanyl, morphine, or lorazepam infusion. For clarity, please comment in the study design section whether these variables were included in the DACAPO database or not.

A: In this post-hoc data retrieval of all DACAPO study centres, only a selection of sedatives and analgetic (including dexmedetomidine) could use for reasons of practicability. We describe this fact in the limitations section (page 14).

4. C: In the results section, report the number of subjects with positive outcomes findings. Specifically, patients with PHQ-9-score ≥5 and PTSS-14-score ≥45.

A: Thank you once again. We explained it more accurately in Table 3.

5. C: The multivariable linear regression results focused on ketamine and midazolam. It would be helpful to report the results for other analgosedatives as a supplementary appendix table.

A: We added this supporting information as Supplement material (Table S1).

6. C: The discussion section of the manuscript might benefit from discussing why the higher prevalence of psychiatric symptoms was detected at 3- and 6-month and not at the 12-month time point.

A: We discussed this fact in our DACAPO study team, but currently it is not clear, why higher cumulative doses increase the risk of psychiatric symptoms in ARDS survivors up to 6 months after ICU discharge. We clarify this fact in the discussion section.

“Currently, it is unknown why ketamine increased the risk of psychiatric symptoms in the short follow-up outcome and not after long-term follow-up.”

7. C: The conclusion recommends that ketamine be used for a short duration. The findings of this evaluation don't support this recommendation. The study didn't investigate whether the increased incidence of psychiatric symptoms was related to the longer versus shorter duration of use. It might not be appropriate to recommend a shorter duration of ketamine use over a longer duration of use. I would suggest rephrasing the conclusion and recommendations based on the findings of this study.

A: We understand your view and modified the conclusion more accurately.

“The administration of ketamine to ICU patients with ARDS should be with caution.” (Abstract) and “The administration of ketamine to ICU patients with ARDS should be with caution or refrained due to the alternative of other sedative and analgesic drugs.” (Conclusions)

8. C: Minor edits (Page 4: Study design: change DACOPO to DACAPO;
   Page 4: Sample: change 1.225 patients to 1,225 patients (comma versus period);
   Page 5: First paragraph. Clarify the number of patients who died after discharge from the ICU. Figure 1 indicates N=161 versus N=158; Page 5: Consider changing "The kind of analgesia and sedation" to "The type of analgesia and sedation"
   Page 5: "For the screening tools, the cut-off values for being at risk of depression were defined as PHQ-9 (≥5) and for PTSD as PTSS-14 [≥45])." The parenthesis in this phrase is confusing.

A: Thank you for the helpful review. We adapted it according to the revision of the manuscript.

9. C: Please update.Page 7: Please reword "Sedation was often increased with clonidine (73.1%) and midazolam (59.0%)". It's not clear if this refers to an increase in depth/level of sedation or dose of sedatives.

A: We explained it correctly.

"Sedation was often deepened with clonidine (73.1%) and/or midazolam (59.0%)."

10. C: Table 3: Please clarify n=84 in the table legend. Nowhere in the manuscript was a discussion around that number of patients. Please include a detailed explanation of how this number was achieved in the manuscript text.

A: We explained this fact more detailed in the results section (page 7): „From 134 patients, where medication was documented during ICU therapy, the values of SF-12, PHQ-9, and PTSS-14 of 84 ARDS survivors could be compared.“

**Reviewer #2:**

Given that 262 respondents were excluded due to unknown doses and duration of sedation and analgesia application, can the author comment on impact on their conclusions? In other words, would documented dosages of sedatives during their ICU treatment in these respondents changed the conclusion that prolonged ketamine use lowered health quality of life scores?

Answer: Thank you for your helpful comment. In connection with reviewer 1, we have described this fact in more detail in the limitations section and in the conclusions. We know about the number of drop-outs and loss of analgosedation data acquirement, which may have resulted in selection bias and limited this study. Due to technical reason (e.g. lack of electronic patient data management system) a registration of analgosedation data was not possible of each DACAPO study centre. But all received analgosedation data of the recruiting study centres were checked twice for plausibility and accuracy from our DACAPO study group.

“Despite our best efforts to follow up each patient, the number of drop-outs and loss of analgosedation data acquirement was rather high, which may have resulted in selection bias limiting this study. In this post-hoc data collection only a selection of sedatives and analgetic could be used for reasons of practicability.”

“The administration of ketamine to ICU patients with ARDS should be with caution.” (Abstract)

**Reviewer #3:**

The data was basically exploratory as the investigators used data from the prospective observational nation‑ wide ARDS study across Germany (DACAPO) to investigate the influence of sedation and analgesia on HRQoL and the risk of psychiatric symptoms in ARDS survivors 3, 6 and 12 months after their discharge from the intensive care unit (ICU). The analysis was routinely performed using univariate and multivariable procedures as noted in the statistical analysis section of the paper. The tables and figures are well formatted and described. The conclusions appear to follow reasonably from the analyses performed. There are some deficiencies noted:

1. Comment (C): Although retrospective and descriptive, the sample size was 134 subjects and some statistical justification should be given for the adequacy of the sample.

A: All patients of the prospective DACAPO study with data on analgosedation were used for this supplementary analysis. Thus, no more than n=134 patients were available. Nevertheless, this sample is sufficient for the first exploratory analyses regarding our additional questions. As a rule of thumb, a minimum of 10-12 cases is needed per variable for a linear regression model. In our multiple linear regression model with eight independent variables, we needed at least 80 cases for the first exploratory analyses. (page 6)

2. In Table 3 missing data is noted especially at six months which is about 26% of the sample in some cases assuming a denominator of 84 at most. The authors need to give this some attention and explain how the missing data issue is addressed, if at all. There is no mention in the statistical analysis section of how this is handled.

A: Missing data were not imputed in this analysis. A patient with missing data was omitted from the respective analysis. We added a statement to the statistical analysis section (page 5 to 6)

We hope that we have been able to answer the questions adequately and thank you for the critical evaluation of our paper.

Kind regards,

Sebastian Blecha

Anaesthesiologist

Department of Anaesthesiology

University Medical Centre Regensburg

Franz-Josef-Strauss-Allee 11

93053 Regensburg

Germany

Phone:	+49 941 944 7801

Facsimile:	+49 941 944-7802

Email:	Sebastian.Blecha@ukr.de
