# Supplementary material for: Association of analgosedation with psychiatric symptoms and health-related quality of life in ARDS survivors: Post hoc analyses of the DACAPO study
Source: PLoS One. 2022 Oct 21;17(10):e0275743. doi: 10.1371/journal.pone.0275743 (PMC9586389; doi:10.1371/journal.pone.0275743)
Supplement: Attachment — Submitted filename: PONE-D~1.DOC [file pone.0275743.s004.DOC]

**PLOS ONE**

##### Association of analgosedation with psychiatric symptoms and health-related quality of life in ARDS survivors: Post hoc analyses of the DACAPO study

--Manuscript Draft--

| **Manuscript Number:** | PONE-D-22-09940R1 |
| --- | --- |
| **Article Type:** | Research Article |
| **Full Title:** | Association of analgosedation with psychiatric symptoms and health-related quality of life in ARDS survivors: Post hoc analyses of the DACAPO study |
| **Short Title:** | Association of analgosedation with psychiatric symptoms and health-related quality of life in ARDS survivors |
| **Corresponding Author:** | Sebastian Blecha  Universitätsklinikum Regensburg: Universitatsklinikum Regensburg Regensburg, GERMANY |
| **Keywords:** | Acute respiratory distress syndrome; analgosedation; depression; Posttraumatic stress disorder; HRQOL |
| **Abstract:** | Background  The acute respiratory distress syndrome (ARDS) is a life-threatening condition with the risk of developing hypoxia and thus requires for invasive mechanical ventilation a long- term analgosedation. Yet, prolonged analgesia and sedation may be a reason for declining health-related quality of life (HRQoL) and the development of psychiatric disorders.  Methods  We used data from the prospective observational nation-wide ARDS study across Germany (DACAPO) to investigate the influence of sedation and analgesia on HRQoL and the risk of psychiatric symptoms in ARDS survivors 3, 6 and 12 months after their discharge from the intensive care unit (ICU). HRQoL was measured with the Physical and Mental Component Scale of the Short-Form 12 Questionnaire (PCS-12, MCS-12). The prevalence of psychiatric symptoms (depression and post-traumatic stress disorder [PTSD]) was assessed using the Patient Health Questionnaire-9 and the Post- Traumatic Stress Syndrome-14. The associations of analgosedation with HRQoL and psychiatric symptoms were investigated by means of multivariable linear regression models.  Results  The data of 134 ARDS survivors (median age [IQR]: 55 [44–64], 67% men) did not show any significant association between analgosedation and physical or mental HRQoL up to 1 year after ICU discharge. Multivariable linear regression analysis (B [95%-CI]) yielded a significant association between symptoms of psychiatric disorders and increased cumulative doses of ketamine up to 6 months after ICU discharge (after 3 months: depression: 0.15 [0.05, 0.25]; after 6 months: depression: 0.13 [0.03, 0.24]  and PTSD: 0.42 [0.04, 0.80)]).  Conclusions  Up to 1 year after ICU discharge, analgosedation did not influence HRQoL of ARDS survivors. Prolonged administration of ketamine during ICU treatment, however, was positively associated with the risk of psychiatric symptoms. The administration of ketamine to ICU patients with ARDS should be with caution. |
| **Order of Authors:** | Sebastian Blecha |
|  | Florian Zeman |
|  | Magdalena Brandl |
|  |  |

|  | Frank Dodoo-Schittko |
| --- | --- |
|  | Susanne Brandstetter |
|  | Christian Karagiannidis |
|  | Christian Apfelbacher |
|  | Thomas Bein |
| **Opposed Reviewers:** |  |
| **Response to Reviewers:** | Thank you for the opportunity to revise our manuscript entitled: “Association of analgosedation with psychiatric symptoms and health-related quality of life in ARDS survivors: Post hoc analyses of the DACAPO study”. We are very grateful for the positive review, helpful suggestions, and comments of the three reviewers and you to improve our paper. We have thoroughly modified the manuscript accordingly. Detailed responses to the reviewers’ comments are listed in the Response letter. |
| **Additional Information:** |  |
| **Question** | **Response** |
| **Financial Disclosure**  Enter a financial disclosure statement that describes the sources of funding for the work included in this submission. Review the [submission guidelines](http://journals.plos.org/plosone/s/submission-guidelines#loc-financial-disclosure-statement) for detailed requirements. View published research articles from [*PLOS ONE*](http://journals.plos.org/plosone/) for specific examples.  This statement is required for submission and **will appear in the published article** if the submission is accepted. Please make sure it is accurate. | The study is funded by the German Ministry of Education and Research (Bundesministerium für Bildung und Forschung, funding number 01GY1340). The funders had no role in study design, data collection and analysis, decision to publish, or preparation of the manuscript |
| **Unfunded studies**  Enter: *The author(s) received no specific funding for this work.*  **Funded studies**  Enter a statement with the following details:   - Initials of the authors who received each award - Grant numbers awarded to each author - The full name of each funder - URL of each funder website - Did the sponsors or funders play any role in the study design, data collection and analysis, decision to publish, or preparation of the manuscript? - **NO** - Include this sentence at the end of your statement: *The funders had no role in study design, data collection and analysis, decision to publish, or preparation of the manuscript.* - **YES** - Specify the role(s) played. |  |
|  |  |

| * typeset |  |
| --- | --- |
| **Competing Interests**  Use the instructions below to enter a competing interest statement for this submission. On behalf of all authors, disclose any [competing interests](http://journals.plos.org/plosone/s/competing-interests) that could be perceived to bias this work—acknowledging all financial support and any other relevant financial or non- financial competing interests.  This statement is required for submission and **will appear in the published article** if the submission is accepted. Please make sure it is accurate and that any funding sources listed in your Funding Information later in the submission form are also declared in your Financial Disclosure statement.  View published research articles from  [*PLOS ONE*](http://journals.plos.org/plosone/) for specific examples. | CK reports personal fees from Maquet, personal fees from Xenios, personal fees from Bayer, non‑financial support from Speaker of the German register of ICUs, grants from the German Ministry of Research and Education, during the conduct of the study. All other authors (SB, FZ, FDS, SuB, MB, CA, and TB) declare no conflict of interest. |
| **NO authors have competing interests**  Enter: *The authors have declared that no competing interests exist.*  **Authors with competing interests**  Enter competing interest details beginning with this statement:  *I have read the journal's policy and the authors of this manuscript have the following competing interests: [insert competing interests here]* |  |
| * typeset |  |
| **Ethics Statement**  Enter an ethics statement for this submission. This statement is required if the study involved:   - Human participants - Human specimens or tissue | The study was approved by the Ethics Committee of the University of Regensburg (file number 13-101-0262) and where necessary by Ethics Committees of the participating study centres. Written informed consent was obtained from caregivers/legal guardians and additionally from patients who had survived ICU. |

| - Vertebrate animals or cephalopods - Vertebrate embryos or tissues - Field research   Write "N/A" if the submission does not require an ethics statement.  General guidance is provided below. Consult the [submission guidelines](http://journals.plos.org/plosone/s/submission-guidelines#loc-guidelines-for-specific-study-types) for detailed instructions. **Make sure that all information entered here is included in the Methods section of the manuscript.** |  |
| --- | --- |

| **Format for specific study types**  **Human Subject Research (involving human participants and/or tissue)**   - Give the name of the institutional review board or ethics committee that approved the study - Include the approval number and/or a statement indicating approval of this research - Indicate the form of consent obtained (written/oral) or the reason that consent was not obtained (e.g. the data were analyzed anonymously)   **Animal Research (involving vertebrate animals, embryos or tissues)**   - Provide the name of the Institutional Animal   Care and Use Committee (IACUC) or other relevant ethics board that reviewed the study protocol, and indicate whether they approved this research or granted a formal waiver of ethical approval   - Include an approval number if one was obtained - If the study involved *non-human primates*, add *additional details* about animal welfare and steps taken to ameliorate suffering - If anesthesia, euthanasia, or any kind of animal sacrifice is part of the study, include briefly which substances and/or methods were applied   **Field Research**  Include the following details if this study involves the collection of plant, animal, or other materials from a natural setting:   - Field permit number - Name of the institution or relevant body that granted permission |  |
| --- | --- |
|  |  |
| **Data Availability**  Authors are required to make all data underlying the findings described fully available, without restriction, and from the time of publication. PLOS allows rare exceptions to address legal and ethical concerns. See the [PLOS Data Policy](http://journals.plos.org/plosone/s/data-availability) and [FAQ](http://journals.plos.org/plosone/s/data-availability#loc-faqs-for-data-policy) for detailed information. | No - some restrictions will apply |

| A Data Availability Statement describing where the data can be found is required at submission. Your answers to this question constitute the Data Availability Statement and **will be published in the article**, if accepted. |  |
| --- | --- |
| **Important:** Stating ‘data available on request from the author’ is not sufficient. If your data are only available upon request, select ‘No’ for the first question and explain your exceptional situation in the text box. |  |
| Do the authors confirm that all data underlying the findings described in their manuscript are fully available without restriction? |  |
| **Describe where the data may be found in full sentences. If you are copying our sample text, replace any instances of XXX with the appropriate details.**   - If the data are **held or will be held in a public repository**, include URLs, accession numbers or DOIs. If this information will only be available after acceptance, indicate this by ticking the box below. For example: *All XXX files are available from the XXX database (accession number(s) XXX, XXX.*). - If the data are all contained **within the manuscript and/or Supporting Information files**, enter the following: *All relevant data are within the manuscript and its Supporting Information files.* - If neither of these applies but you are able to provide **details of access elsewhere**, with or without limitations, please do so. For example:   *Data cannot be shared publicly because of [XXX]. Data are available from the XXX Institutional Data Access / Ethics Committee (contact via XXX) for researchers who meet the criteria for access to confidential data.*  *The data underlying the results presented in the study are available from (include the name of the third party* | The study data cannot be shared publicly because the written consent obtained from the study participants did not include a public availability of their data. Data are available from the two principal investigators (PIs) Christian Apfelbacher and Thomas Bein (E-mail: [Christian.Apfelbacher@med.ovgu.de](mailto:Christian.Apfelbacher@med.ovgu.de) and/or Thomas.Bein@ukr.de) on request. |

| *and contact information or URL).*   - This text is appropriate if the data are owned by a third party and authors do not have permission to share the data.   * typeset |  |
| --- | --- |
| Additional data availability information: |  |

Cover Letter

Sebastian Blecha, M.D. Department of Anaesthesiology

University Medical Centre Regensburg Franz-Josef-Strauss-Allee 11

93053 Regensburg Germany

Dear Mrs. Linda L. Maerz,

Thank you for the opportunity to revise our manuscript entitled: “Association of analgosedation with psychiatric symptoms and health-related quality of life in ARDS survivors: Post hoc analyses of the DACAPO study”. We are very grateful for the positive review, helpful suggestions, and comments of the reviewers and you to improve our manuscript. We have thoroughly modified the manuscript accordingly.

This prospective nationwide study investigated the impact of a prolonged analgosedation on health-related quality of life (HRQoL) and the development of psychiatric disorders in a cohort of 134 ARDS survivors. The cohort was recruited within the DACAPO study (“DACAPO: Surviving ARDS: the influence of quality of care and individual patient characteristics on quality of life” funded by the German Federal Ministry of Education and Research).

We found that analgosedation did not influence HRQoL of ARDS survivors, but the multivariable linear regression analysis established the cumulative dose of ketamine as a significant predictor for depression and PTSD in ARDS survivors up to 6 months after ICU discharge. The association between sedation and patient-reported psychiatric outcomes is novel and could influence the long-term sedation regime of ICU patients with ARDS.

We hope that this paper merits publication in the Journal “PLOS ONE”. For all authors

PD Dr. Sebastian Blecha

Prof. Dr. Thomas Bein

Prof. Dr. Christian Karagiannidis

Manuscript	[Click here to access/download;Manuscript;Manuscript-090822- PLoS One.docx](https://www.editorialmanager.com/pone/download.aspx?id=31645574&guid=9f4ce222-d316-4e40-b6ed-5488956b4383&scheme=1)

# Association of analgosedation with psychiatric symptoms and health-related quality of life in ARDS survivors: Post hoc analyses of the DACAPO study

Sebastian Blecha^1*^, [Sebastian.Blecha@ukr.de](mailto:Sebastian.Blecha@ukr.de) Florian Zeman^2^, [Florian.Zeman@ukr.de](mailto:Florian.Zeman@ukr.de)

Magdalena Rohr^3^, [Magdalena.Rohr@klinik.uni-regensburg.de](mailto:Magdalena.Rohr@klinik.uni-regensburg.de)

Frank Dodoo-Schittko^3,#a^, [Frank.Dodoo-Schittko@klinik.uni-regensburg.de](mailto:Frank.Dodoo-Schittko@klinik.uni-regensburg.de) Susanne Brandstetter^3,#b^, [Susanne.Brandstetter@klinik.uni-regensburg.de](mailto:Susanne.Brandstetter@klinik.uni-regensburg.de) Christian Karagiannidis^4^, [karagiannidisc@kliniken-koeln.de](mailto:karagiannidisc@kliniken-koeln.de)

Christian Apfelbacher^3,#a^, [Christian.Apfelbacher@med.ovgu.de](mailto:Christian.Apfelbacher@med.ovgu.de) Thomas Bein^5^, [Thomas.Bein@ukr.de](mailto:Thomas.Bein@ukr.de)

for the DACAPO study group

1 Department of Anaesthesiology, University Medical Centre Regensburg, Regensburg, Germany
2 Centre of Clinical Studies, University Medical Centre Regensburg, Regensburg, Germany
3 Medical Sociology, Institute of Epidemiology and Preventive Medicine, University of Regensburg, Regensburg, Germany
4 Department of Pneumology and Critical Care Medicine, Cologne-Merheim Hospital, ARDS and ECMO Centre, Kliniken der Stadt Köln gGmbH, Witten/Herdecke University Hospital, Cologne, Germany
5 Faculty of Medicine, University of Regensburg, Regensburg, Germany

#a	Current Address: Institute of Social Medicine and Health Systems Research, Otto-von-

Guericke-University Magdeburg, Magdeburg, Germany

#b	Current Address: University Children‘s Hospital Regensburg (KUNO), University of Regensburg, Regensburg, Germany

*Corresponding author:

Email: [Sebastian.Blecha@ukr.de](mailto:Sebastian.Blecha@ukr.de)

Trial registration:

Clinicaltrials.gov: NCT02637011 (Registered 15 December 2015, retrospectively registered)

**Word count:** 2436

# Abstract

**Background:** The acute respiratory distress syndrome (ARDS) is a life-threatening condition with the risk of developing hypoxia and thus requires for invasive mechanical ventilation a long-term analgosedation. Yet, prolonged analgosedation may be a reason for declining health- related quality of life (HRQoL) and the development of psychiatric disorders.

**Methods:** We used data from the prospective observational nation‑ wide ARDS study across Germany (DACAPO) to investigate the influence of sedation and analgesia on HRQoL and the risk of psychiatric symptoms in ARDS survivors 3, 6 and 12 months after their discharge from the intensive care unit (ICU). HRQoL was measured with the Physical and Mental Component Scale of the Short‑ Form 12 Questionnaire (PCS‑ 12, MCS‑ 12). The prevalence of psychiatric symptoms (depression and post‑ traumatic stress disorder [PTSD]) was assessed using the Patient Health Questionnaire‑ 9 and the Post‑ Traumatic Stress Syndrome‑ 14. The associations of analgosedation with HRQoL and psychiatric symptoms were investigated by means of multivariable linear regression models.

**Results:** The data of 134 ARDS survivors (median age [IQR]: 55 [44–64], 67% men) did not show any significant association between analgosedation and physical or mental HRQoL up to 1 year after ICU discharge. Multivariable linear regression analysis (B [95%‑ CI]) yielded a significant association between symptoms of psychiatric disorders and increased cumulative doses of ketamine up to 6 months after ICU discharge (after 3 months: depression: 0.15 [0.05, 0.25]; after 6 months: depression: 0.13 [0.03, 0.24] and PTSD: 0.42 [0.04, 0.80)]).

**Conclusions:** Up to 1 year after ICU discharge, analgosedation did not influence HRQoL of ARDS survivors. Prolonged administration of ketamine during ICU treatment, however, was positively associated with the risk of psychiatric symptoms. The administration of ketamine to ICU patients with ARDS should be with caution.

**Keywords:** Acute respiratory distress syndrome, analgosedation, depression, posttraumatic stress disorder, HRQoL

# Background

The acute respiratory distress syndrome (ARDS) is a life-threatening condition, which is characterised by either direct or indirect damage to the lung parenchyma, often resulting in critical hypoxemia or hypercapnia, or both [1]. In the initial phase of ARDS, patients require long-term analgosedation for invasive mechanical ventilation. In the current context, patients with severe COVID-19-associated ARDS are usually ventilated for a mean duration of 13 to 16 days [2,3]. Prolonged sedation during intensive care therapy may be a potential risk factor for the development of psychiatric disorders and delirium [4,5].

Additionally, ARDS survivors have a substantial risk of developing anxiety, depression and symptoms of posttraumatic stress disorder (PTSD) as well as of decreased health-related quality of life (HRQoL)[6,7]. A recent study of 114 COVID-19-associated ARDS survivors in Italy showed symptoms of depression in 9% of patients, of anxiety in 10% and of PTSD in 4% [8]. A study of 113 COVID-19-associated ARDS survivors in Spain showed that over 90% of patients had developed one or more symptoms of a mental disorder [9].

In the current study, we assessed the type and dosage of sedation and analgesia administered during intensive care treatment and the influence of analgosedation on HRQoL and psychiatric disorders up to 1 year after ARDS survival. We hypothesised that analgesics and sedation drugs and their administered doses during ICU treatment increased the risk of psychiatric symptoms and reduced HRQoL of long-term survivors of ARDS.

# Methods

### Study design

The extent and duration of analgosedation and co-medications of ARDS survivors was recorded in the context of a large prospective nation-wide cohort study across Germany (DACAPO study, ClinicalTrials.gov Identifier: NCT02637011) [10]. The DACAPO study was focused on the influence of the quality of care and individual patient characteristics on HRQoL and the time point of returning to work of ARDS survivors. Ethical approval was obtained from the ethics committee of the University of Regensburg (file number: 13–101-0262) and additionally from the ethics committees overseeing the respective study sites [11]. The baseline characteristics and profile of the cohort have been described in more detail elsewhere [12]. ARDS survivors were asked to complete comprehensive self-report questionnaires 3, 6 and 12 months after their discharge from the intensive care unit (ICU).

### Sample

**Fig. 1** gives an overview of the sample size at different time points of the study. Written informed consent was obtained from 1,225 patients with ARDS, who had been treated at one of 61 ICUs across Germany between September 2014 and April 2016. The study included adults with the diagnosis of ARDS (according to the Berlin definition [13]). Patients or their caregivers or legal guardians were approached during the patients’ ICU stay and asked to provide written informed consent. In cases in which caregivers or legal guardians consented to a patient’s participation in the study, the patient had to confirm this preliminary consent at discharge from the ICU.

Out of 877 ICU survivors, 396 (45.2%) had returned the questionnaire at 12 months. The most frequent reason for dropping out of the study was death after discharge from the ICU (N=161). Other reasons included the inability to complete the questionnaire (insufficient knowledge of

German or incapability due to morbidity), absence of a person who could provide proxy reports, withdrawal of consent or an invalid address. Furthermore, the doses and duration of analgosedation could not be assessed in 262 ARDS survivors.

### Data collection and measuring instruments

Characteristics of patient disease and treatment as well as information on ICU discharge were reported by study physicians or physicians from the participating ICUs using the electronic data capture system OpenClinica (OpenClinica, LLC; https:/[/www.openclinica.com/).](http://www.openclinica.com/)) The type of analgesia and sedation, duration and cumulative doses of drugs were extracted and transmitted from the patient data management system of nine recruiting study centres (8 University hospitals and one hospital for basic and standard care). Information on HRQoL and psychiatric disorders was assessed by means of self-report questionnaires at 3, 6 and 12 months after discharge from the ICU. ARDS survivors completed the Short Form-12 self-report questionnaire (SF-12) as a measure of HRQoL and questionnaires on depressive symptoms and post-traumatic stress disorder (PTSD). The Patient Health Questionnaire-9 [PHQ-9] was used to detect symptoms of depression and the Post-Traumatic Stress Syndrome 14-Questions Inventory [PTSS-14] to detect PTSD [14]. For the screening tools, the cut-off values for being at risk of depression were defined as PHQ-9 ≥5 and for PTSD as PTSS-14 ≥45 [15,16]. The published scoring algorithm of SF-12 resulted in the Physical Component Summary (PCS-12) and the Mental Component Summary (MCS-12) scores [2]. Scores range from 0 to 100 (higher values indicate better HRQoL); a score of 50 represents the mean value for the general population (German norm values [17,18]).

### Statistical analyses

All patients of the prospective DACAPO study with data on analgosedation were used for this supplementary analysis. Thus, no more than n=134 patients were available. Nevertheless, this sample is sufficient for the first exploratory analyses regarding our additional questions. As a

rule of thumb, a minimum of 10-12 cases is needed per variable for a linear regression model. In our multiple linear regression model with eight independent variables, we needed at least 80 cases for the first exploratory analyses. Data are shown as mean ± SD or median (IQR) for continuous variables and as absolute and relative frequencies for categorical variables. To assess which analgosedation has a potential impact on HRQoL and the risk of psychiatric symptoms after 3, 6 and 12 months, bivariate models (including type of analgosedation yes/no plus cumulative doses) were calculated for each type of analgosedation and each endpoint. For each endpoint, all significant results were included in a multiple linear regression model, which was further adjusted for the known risk factors age, sex, Simplified Acute Physiology Score II (SAPS II) and length of the ICU stay. The regression coefficient B for the linear regression models was calculated as effect estimate accompanied by the corresponding 95%-confidence interval (95%-CI). A p-value <0.05 was considered significant. Due to the exploratory nature of this study, no adjustments for multiple comparisons were used. All analyses were performed using the software R (Version 3.5.1, [www.r-project.org](http://www.r-project.org/)).

# Results

Out of 396 responding ARDS survivors, 134 patients were analysed 12 months after their ICU discharge (**Figure 1**). The patient characteristics are shown in **Table 1**. Median age at ICU admission was 55 years, two-thirds of the 12-month respondents were men and median length of the ICU stay was 23.5 days. The majority of patients had moderate or severe ARDS (82.1%). Tracheostomy was performed in 46% of ARDS survivors after a median time of 11 days (IQR: 7–16).

**Table 1:** Patient characteristics of ARDS survivors with documented analgosedation (n=134)

| **Patient characteristics** |  | **Missing** (n) |
| --- | --- | --- |
| **Sex** | | |
| Men, n (%) | 90 (67) | 0 |
| Women, n (%) | 44 (33) |  |
| **Age,** years, median (IQR) | 55 (44-64) | 0 |
| **BMI (kg/m²)**, median (IQR) | 26 (24-30) | 0 |
| **Severity of ARDSa** | | |
| Mild, n (%) | 24 (17.9) | 0 |
| Moderate, n (%) | 75 (56.0) |  |
| Severe, n (%) | 35 (26.1) |  |
| **SAPSb,** median (IQR) | 38 (30-46) | 5 |
| **SOFA scorea,** median (IQR) | 8 (6-11) | 9 |
| **Duration of ICU stay,**  days, median (IQR) | 23.5 (15-35) | 0 |
| **Tracheotomy during ICU stay** | | |
| Yes, n (%) | 61 (46) | 1 |
| No, n (%) | 72 (54) |  |

Notes: ARDS: acute respiratory distress syndrome, BMI: body mass index, ICU: intensive care unit, SAPS: Simplified Acute Physiology Score, SOFA: Sequential Organ Failure Assessment; ^a^measured at the time of ARDS diagnosis, ^b^measured 24 hours after ICU admission

The drugs used for analgesia and sedation during ICU treatment in ARDS survivors are shown in **Table 2**. In the following, the number of sedative and analgesic agents that patients received during ICU treatment is listed (based on Table 2): 12 patients (8.9%) got a sixfold, 28 patients (20.9%) fivefold, 38 patients (28.4%) a fourfold, 34 patients (25.4%) a threefold, 19 patients (14.2%) twofold and only 3 patients (2.2%) single analgosedation. The most common type of analgosedation was the combination of intravenously administered propofol and sufentanil. Sedation was often deepened with clonidine (73.1%) and/or midazolam (59.0%). Ketamine or esketamine was administered in 32.1% of patients. Thirty-two of 39 patients (82.1%) with ketamine application and all patients with esketamine additionally received midazolam. Inhalative sedation with isoflurane administered via the AnaConDa® system was used in only 6% of patients.

**Table 2:** Total cumulative dose and duration of sedation and analgesia in ARDS survivors (N=134)

| **Sedation and analgesic agents** | **n (**%**)** | **Total cumulative dose**  mg, median (IQR) | **Duration of sedation and analgesia**  days, median (IQR) |
| --- | --- | --- | --- |
| **Propofol** | 129 (96.3) | 21355 (11786–39177) | 7 (4–10) |
| **Sufentanil** | 124 (92.5) | 5121 (23–18386) | 12 (7–20) |
| **Midazolam** | 79 (59.0) | 1084 (295–4100) | 6 (3–14) |
| **Ketamine** | 39 (29.1) | 8830 (2247–31034) | 3 (1–7) |
| **Esketamine** | 4 (3.0) | 5225 (164–10260) | 5 (2–8) |
| **Clonidine** | 98 (73.1) | 7 (3–20) | 5 (2–10) |
| **Dexmedetomidine** | 20 (14.9) | 0.21 (0.03–2.62) | 4 (2–8) |
| **Isoflurane** | 8 (6.0) | - | 8 (4–15) |

HRQoL and symptoms of psychiatric disorders of ARDS survivors 3, 6 and 12 months after ICU discharge are shown in **Table 3**. From 134 patients, for whom medication was documented during ICU therapy, the values of SF-12, PHQ-9, and PTSS-14 of 84 ARDS survivors could be compared. According to SF-12 measurement, ARDS survivors had lower HRQoL (<50) across all follow-up time points compared to the general population norm [18]. Additionally, the mean increased PHQ-9 values show symptoms of depression across all follow-up time points. 20.2% of patients reported symptoms of PTSD (cut-off scores for symptoms of PTSD: ≥45) and 54.2

% symptoms of depression (PHQ-9 score ≥5) at the 1-year follow-up, respectively.

**Table 3**: HRQoL and symptoms of psychiatric disorders in ARDS survivors after 3, 6 and 12 months (N=84)

|  |  | | **Missing** (n) |
| --- | --- | --- | --- |
| **3 months after ICU discharge** | | | |
| **PCS-12a,** mean (±SD); ^#^ n (%) | 36.6 (±9.5) ^#^ | ^#^ 64 (87.7) | 11 |
| **MCS-12a,** mean (±SD); ^#^ n (%) | 43.7 (±12.1) ^#^ | ^#^ 46 (63.0) | 11 |
| **PHQ-9a,** mean (±SD); *n (%) | 5.6 (±4.7)* | * 38 (45.8) | 1 |
| **PTSS-14a,** mean (±SD); *n (%) | 29.1 (±13.5) | * 12 (14.5) | 1 |
| **6 months after ICU discharge** | | | |
| **PCS-12a,** mean (±SD); ^#^ n (%) | 40.2 (±11.8) ^#^ | ^#^ 47 (75.8) | 22 |
| **MCS-12a,** mean (±SD); ^#^ n (%) | 42.1 (±14.4) ^#^ | ^#^ 40 (64.5) | 22 |
| **PHQ-9a,** mean (±SD); *n (%) | 6.1 (±5.1)* | * 45 (54.2) | 1 |
| **PTSS-14a,** mean (±SD); *n (%) | 32.2 (±17.7) | * 16 (20.0) | 4 |
| **12 months after ICU discharge** | | | |
| **PCS-12a,** mean (±SD); ^#^ n (%) | 42.7 (±11.7) ^#^ | ^#^ 46 (63.9) | 12 |
| **MCS-12a,** mean (±SD); ^#^ n (%) | 45.6 (±13.9) ^#^ | ^#^ 38 (52.8) | 12 |
| **PHQ-9a,** mean (±SD); *n (%) | 5.8 (±5.2)* | * 45 (54.2) | 1 |
| **PTSS-14a,** mean (±SD); *n (%) | 31.5 (±16.8) | * 17 (20.2) | 0 |

Notes: MCS-12: mental component scale of short-form 12 questionnaire; PCS-12: physical component scale of short-form 12 questionnaire; PTSD: post-traumatic stress disorder, PHQ- 9: Patient Health Questionnaire-9, PTSS-14: Post-Traumatic Stress Syndrome 14-Questions Inventory, ^a^health-related quality of life and symptoms of psychiatric disorders (depression, PTSD) were assessed according to the results of patient self-reported questionnaires; # PCS- 12<50 and MCS-12<50 represents worse HRQoL compared to that of the general (German) population; *cut-off values for being at risk of depression and PTSD: PHQ-9-score ≥5 and PTSS-14-score ≥45

Analysing the impact of analgosedation of all given drugs (propofol, midazolam, sufentanil, [es-]ketamine, clonidine, dexmedetomidine and isoflurane) on HRQoL and symptoms of psychiatric disorders during the follow-up time points, only the cumulative doses of ketamine and midazolam showed a significant linear relationship to PHQ-9 and PTSS-14 in the bivariable linear regression models. The relationship of ketamine to PHQ-9 and PTSS-14 scores is shown in **Fig. 2** and that of midazolam to PHQ-9 and PTSS-14 scores in **Fig. 3**. HRQoL (PCS- 12/MCS-12) was not affected by analgesia and sedation at any follow-up time point.

Multivariable linear regression analysis only established the cumulative dose of ketamine as a significant predictor for depression and PTSD (**Table 4 and S1**). Higher doses of ketamine increased the risk of psychiatric symptoms in ARDS survivors up to 6 months after ICU discharge. The number of drugs for analgosedation showed no influence on the psychiatric outcome in the multivariable linear regression analysis.

**Table 4:** Multiple linear regression models of the influence of cumulative ketamine doses on psychiatric symptoms in ARDS survivors 3, 6 and 12 months after ICU discharge

|  | **PHQ-9a** | | **PTSS-14a** | |
| --- | --- | --- | --- | --- |
| **Independent variables#** | **B (95%-CI)** | **P-value** | **B (95%-CI)** | **P-value** |
|  | **3 months after ICU discharge** | | | |
| Ketamine | –1.5 (-4.3, 1.3) | 0.297 | –0.12 (–7.74, 7.50) | 0.975 |
| Cumulative ketamine dose** | 0.15 (0.05, 0.25) | **0.004*** | 0.24 (–0.05, 0.53) | 0.102 |
| Midazolam | 0.60 (–1.83, 3.02) | 0.625 | 2.53 (–4.27, 9.33) | 0.461 |
| Cumulative midazolam dose** | –0.09 (–0.61, 0.44) | 0.742 | 0.96 (–0.69, 2.61) | 0.248 |
|  | **6 months after ICU discharge** | | | |
| Ketamine | –2.8 (–5.8, 0.19) | 0.066 | –9.94 (–20.86, 0.99) | 0.074 |
| Cumulative ketamine dose** | 0.13 (0.03, 0.24) | **0.014*** | 0.42 (0.04, 0.80) | **0.029*** |
| Midazolam | –2.0 (–4.7, 0.67) | 0.138 | –2.65 (–12.31, 7.02) | 0.587 |
| Cumulative midazolam dose** | 0.29 (–0.20, 0.78) | 0.241 | 0.63 (–1.14, 2.40) | 0.479 |
|  | **12 months after ICU discharge** | | | |

| Ketamine | –2.68 (–5.97, 0.62) | 0.110 | –8.58 (–19.53, 2.38) | 0.123 |
| --- | --- | --- | --- | --- |
| Cumulative ketamine dose** | 0.03 (–0.08, 0.15) | 0.572 | 0.15 (–0.24, 0.53) | 0.453 |
| Midazolam | 0.04 (–2.80, 2.88) | 0.978 | –0.62 (–10.00, 8.75) | 0.895 |
| Cumulative midazolam dose** | 0.09 (–0.47, 0.64) | 0.761 | 0.46 (–1.36, 2.29) | 0.616 |

Notes: *p <0.05; **per 1000 mg; ^#^further adjusted for age, sex, SAPS, days on ICU; B: regression coefficient, 95%-CI: 95% confidence interval, PHQ-9: Patient Health Questionnaire- 9; PTSS-14: Post-Traumatic Stress Syndrome 14-Questions Inventory; ^a^symptoms of psychiatric disorders (depression and PTSD) were diagnosed according to the results of patient self-reported questionnaires

# Discussion

This study analysed the influence of sedation and analgesia on HRQoL and the prevalence of psychiatric symptoms in 134 ARDS survivors up to 1 year after ICU discharge. The main findings were: 1) Increased cumulative ketamine doses for analgosedation during ICU treatment were significantly associated with a higher prevalence of psychiatric symptoms 3 and 6 months after ICU discharge and 2) Analgosedation was not significantly associated with physically (PCS-12) or mentally impaired quality of life (MCS-12).

##### Analgosedation during ICU treatment

At the onset of ARDS, deep analgosedation is often needed. After initial stabilisation during the recovery phase, a protocol-based sedation regimen emphasising light or no sedation, daily spontaneous awakening and breathing periods as well as physiotherapy may improve short- term outcome and decrease long-term complications including the risk of cognitive decline and psychiatric symptoms [19,20].

In our study, the most common type of analgosedation during ICU treatment was the combination of intravenously administered propofol and sufentanil, often supplemented or replaced with midazolam, clonidine or dexmedetomidine. Ketamine was administered in nearly one third of the patients. In a recent study by Ren and colleagues, all 42 patients with ARDS

who received venovenous extracorporeal membrane oxygenation were given midazolam combined with sufentanil or remifentanil; in 69%, analgosedation was supplemented with propofol and in 25% with dexmedetomidine [21].

##### HRQoL and psychiatric symptoms in ARDS survivors

The literature for analysing the association between analgosedation and the development of psychiatric symptoms in ARDS survivors is very scarce. Stevenson and colleagues found that 38% of 152 ARDS survivors had a positive screening test for general anxiety 3 months after ICU discharge, and pre-ICU body mass index and psychiatric comorbidity were negatively associated with the development of general anxiety, but without any association of daily administered analgesics, sedatives and steroids with general anxiety symptoms [22]. Bienvenu et al. showed in a 5-year follow-up study that generally 32% of ARDS survivors had prolonged symptoms of depression, 38% of anxiety and 23% of PTSD [6]. Furthermore, the Physical and Mental Component Score often shows decreased HRQoL in ARDS survivors in the long-term follow up [23]. In our study, ARDS survivors had lower HRQoL measured with the PSC-12 and MCS-12 at each follow-up time point compared to the score validated from the general population [17]. Except for ketamine, analgosedation had no impact on HRQoL or the development of psychiatric symptoms. In this post hoc-analysis, 20.2% of patients had symptoms of PTSD and 54.2 % symptoms of depression at the 1-year follow-up.

##### Indications and risks of ketamine and midazolam administration

Ketamine is an established sedative with a unique mechanism of action and side effect profile. The primary mechanism of action is antagonism of the N-methyl-D-aspartate receptor, which appears to be responsible for the amnestic and analgesic effects. In addition, ketamine stimulates noradrenergic neurons and inhibits catecholamine uptake, which may preserve cardiovascular stability. Ketamine also enhances the descending inhibiting serotoninergic pathway and exerts anti-depressive effects [24]. This is the reason why ketamine is commonly

used in patients with major depressive disorders, bipolar depression and treatment-resistant depression [25–27].

A recent prospective study including more than 1000 critically ill surgical and medical patients requiring analgosedation suggested that ketamine may also increase the risk of incident delirium [28]. However, another study including 172 ICU patients showed adjunctive continuous infusion with ketamine to be safe and effective for sedation without any increased risk of delirium. Additionally, in that study, ketamine was associated with reduced norepinephrine requirement, days of benzodiazepine administration and continuous opioid infusion [29]. In our study, higher cumulative doses of ketamine increased the risk of psychiatric symptoms in ARDS survivors up to 6 months after ICU discharge. Currently, it is unknown why ketamine increased the risk of psychiatric symptoms in the short follow-up outcome and not in the long- term follow-up.

For decades, midazolam as a γ-aminobutyric acid (GABA) receptor agonist has been one of the most commonly administered sedative drugs for ICU patients [30,31]. However, a benzodiazepine dominant sedation strategy (e.g. with midazolam) is associated with an increased risk of developing delirium, prolonged duration of invasive mechanical ventilation and ICU length of stay [13,32,33]. ICU delirium is common and may increase mortality risk and contribute to long-term cognitive impairment [34,35]. In our study, higher cumulative doses of midazolam did not affect the HRQoL and risk of psychiatric symptoms in ARDS survivors.

###### Strengths and limitations

The strengths of the present study are its prospective design with three follow-up time points, the large number of included patients with ARDS from hospitals across Germany and the detailed collection of data on HRQoL, psychiatric symptoms and individual patient characteristics. Despite our best efforts to follow up each patient, the number of drop-outs and

loss of analgosedation data acquirement was rather high, which may have resulted in selection

bias limiting this study. In this post-hoc data collection only a selection of sedatives and analgetic could be used for reasons of practicability. The instruments used for screening mental disorders do not allow making diagnoses such as major depression disorders and PTSD; thus, only symptoms or the risk of being affected by such a disorder were recorded.

# Conclusion

In summary, prolonged administration of ketamine during ICU treatment was positively associated with the risk of psychiatric symptoms. The analgesia and sedation did not influence HRQoL of ARDS survivors up to 1 year after ICU discharge. The administration of ketamine to ICU patients with ARDS should be with caution or refrained due to the alternative of other sedative and analgesic drugs. Larger prospective studies are needed to analyse the influence of analgosedation and notably of ketamine during ICU treatment on the development of psychiatric symptoms in ARDS survivors.

# Declarations

### List of abbreviations

ARDS: Acute respiratory distress syndrome; BMI: Body mass index; HRQoL: Health‑ related quality of life; ICU: Intensive care unit; MCS‑ 12: Mental Component Summary; PCS‑ 12: Physical Component Summary; PHQ‑ 9: Patient Health Questionnaire‑ 9; PTSD: Posttraumatic stress disorders; PTSS‑ 14: Post‑ Traumatic Stress Syndrome 14‑ Questions Inventory; SAPS II: Simplified Acute Physiology Score; SF ‑ 12: Short Form‑ 12 self‑ report questionnaire; SOFA: Sequential organ failure assessment

### Ethics approval and consent to participate

The study was approved by the Ethics Committee of the University of Regensburg (file number 13-101-0262) and where necessary by Ethics Committees of the participating study centres. Written informed consent was obtained from caregivers/legal guardians and additionally from patients who had survived ICU.

### Consent for publication

Not applicable

### Availability of data and materials

The study data cannot be shared publicly because the written consent obtained from the study participants did not include a public availability of their data. Data are available from the two principal investigators (PIs) Christian Apfelbacher and Thomas Bein (E-mail: [Christian.Apfelbacher@med.ovgu.de](mailto:Christian.Apfelbacher@med.ovgu.de) and/or [Thomas.Bein@ukr.de](mailto:Thomas.Bein@ukr.de)) on request.

### Conflict of interest

CK: I have read the journal's policy and the authors of this manuscript have the following competing interests: personal fees from Maquet, personal fees from Xenios, personal fees from Bayer, non‑ financial support from Speaker of the German register of ICUs, grants from the German Ministry of Research and Education, during the conduct of the study. All other authors (SB, FZ, FDS, SuB, MB, CA, and TB) declare no conflict of interest.

### Funding

The study was funded by the German Ministry of Education and Research (Bundesministerium für Bildung und Forschung, funding number 01GY1340, [https://www.gesundheitsforschung-](https://www.gesundheitsforschung-bmbf.de/de/versorgungsstudien-3401.php)

[bmbf.de/de/versorgungsstudien-3401.php,](https://www.gesundheitsforschung-bmbf.de/de/versorgungsstudien-3401.php) principal investigators CA and TB). The funders had no role in study design, data collection and analysis, decision to publish, or preparation of the manuscript.

### Author Contributions

**Conceptualization:** Sebastian Blecha, Magdalena Rohr, Frank Dodoo-Schittko, Susanne Brandstetter, Christian Karagiannidis, Christian Apfelbacher, Thomas Bein

**Data curation:** Sebastian Blecha, Magdalena Rohr, Susanne Brandstetter, Christian Karagiannidis,

**Formal analysis:** Sebastian Blecha, Florian Zeman, Magdalena Rohr

**Investigation:** Sebastian Blecha, Magdalena Rohr, Christian Karagiannidis

**Methodology:** Sebastian Blecha, Florian Zeman, Magdalena Rohr, Frank Dodoo-Schittko, Susanne Brandstetter, Christian Karagiannidis, Christian Apfelbacher, Thomas Bein

**Project administration:** Christian Apfelbacher, Thomas Bein

**Resources:** Sebastian Blecha, Magdalena Rohr, Christian Karagiannidis, Christian Apfelbacher, Thomas Bein

**Software:** Florian Zeman, Magdalena Rohr

**Supervision:** Susanne Brandstetter, Frank Dodoo-Schittko, Christian Karagiannidis, Christian Apfelbacher, Thomas Bein

**Validation:** Sebastian Blecha, Florian Zeman, Magdalena Rohr, Susanne Brandstetter

**Visualization:** Sebastian Blecha, Florian Zeman

**Writing – original draft:** Sebastian Blecha

**Writing – review & editing:** Florian Zeman, Magdalena Rohr, Frank Dodoo-Schittko, Susanne Brandstetter, Christian Karagiannidis, Christian Apfelbacher, Thomas Bein

# Acknowledgement

We are indebted to the highly committed intensivists and study assistants across Germany who had recruited patients for the DACAPO study:

Aachen, Aachen University Hospital RWTH Aachen, Department of Anaesthesiology (PD Dr. Johannes Bickenbach, Dr. Thorben Beeker, PD Dr. Tobias Schürholz, Jessica Pezechk);

Amberg, Klinikum Amberg, Department for Anaesthesiology (Dr. Jens Schloer); Augsburg, Klinikum Augsburg (Dr. Ulrich Jaschinski, Ilse Kummer); Bamberg, Sozialstiftung Bamberg Hospital, Department for Anaesthesiology (Dr. Oliver Kuckein); Berlin, Charité - University Medicine Berlin, Department of Anaesthesiology and Intensive Care Medicine (PD Dr. Steffen Weber-Carstens, Dr. Anton Goldmann, Dr. Stefan Angermair, Krista Stoycheva); Berlin, HELIOS Klinikum Berlin-Buch, Department of Intensive Care Medicine (Prof. Dr. Jörg Brederlau, Nadja Rieckehr, Gabriele Schreiber, Henriette Haennicke); Bielefeld, Ev. Krankenhaus Bielefeld. Department of Anaesthesiology, Intensive Care Medicine, Emergency Medicine and Pain Therapy (Dr. Friedhelm Bach, Dr. Immo Gummelt, Dr. Silke Haas, Catharina Middeke, Dr. Ina Vedder, Marion Klaproth); Bochum, Ruhr University Bochum, Department of Anaesthesiology (Prof. Dr. Michael Adamzik, Dr. Jan Karlik, Dr. Stefan Martini, Luisa Robitzky); Bonn, University Hospital Bonn, Department of Anaesthesiology and Intensive Care Medicine (Prof. Dr. Christian Putensen, Dr. Thomas Muders, Ute Lohmer); Bremen, Klinikum Bremen-Mitte, Department of Anaesthesiology (Prof. Dr. Rolf Dembinski); Deggendorf, Medical Centre, Department of Anaesthesiology and Intensive Care Medicine (Dr. Petra Schäffner, Dr. Petra Wulff-Werner); Dortmund, Klinikum Dortmund, Department of Critical Care Medicine (Elke Landsiedel-Mechenbier, Daniela Nickoleit-Bitzenberger, Ann- Kathrin Silber); Dresden, University Hospital Dresden Carl Gustav Carus, Department of Anaesthesiology and Intensive Care Medicine (Prof. Dr. Maximilian Ragaller, Prof. Dr. Marcello Gama de Abreu, Alin Ulbricht, Linda Reisbach); Frankfurt am Main, University Hospital Frankfurt, Department of Anaesthesiology, Intensive Care Medicine and Pain Therapy (Prof. Dr. Kai Zacharowski, Prof. Dr. Patrick Meybohm, Simone Lindau, Haitham Mutlak); Freiburg, University Medical Center Freiburg, Department of Anaesthesiology and Critical Care Medicine Freiburg (Prof. Dr. Alexander Hötzel, Dr. Johannes Kalbhenn); Freising, Klinikum Freising, Department of Anaesthesiology (Dr. Christoph Metz, Dr. Stefan Haschka); Göppingen, Klinik am Eichert, ALB FILS Kliniken, Department of Anaesthesiology and Intensive Care (Dr. Stefan Rauch); Göttingen, University Medical Center, Department of Anaesthesiology, Emergency and Intensive Care Medicine (Prof. Dr. Michael Quintel, Dr. Lars-Olav Harnisch, Dr. Sophie Baumann, Andrea Kernchen); Greifswald, University Medicine Greifswald, Department of Internal Medicine B (Dr. Sigrun Friesecke, Sebastian Maletzki); Hamburg, University Hospital Hamburg-Eppendorf, Department of Intensive Care Medicine, Centre of Anaesthesiology and Intensive Care Medicine (Prof. Dr. Stefan Kluge, Dr. Olaf Boenisch, Dr. Daniel Frings, Birgit Füllekrug, Dr. Nils Jahn, Dr. Knut Kampe, Grit Ringeis, Brigitte Singer, Dr. Robin Wüstenberg); Hannover, Hannover Medical School,

Department of Anaesthesiology and Intensive Care Medicine (Dr. Jörg Ahrens, Dr. Heiner Ruschulte, Dr. Andre Gerdes, Dr. Matthias Groß); Hannover, Hannover Medical School, Department of Respiratory Medicine (Dr. Olaf Wiesner, Aleksandra Bayat-Graw); Heidelberg, University of Heidelberg, Department of Anaesthesiology (Dr. Thorsten Brenner, Dr. Felix Schmitt, Anna Lipinski) ; Herford, Klinikum Herford, Clinic for Anaesthesiology, Surgical Intensive Care Medicine, Emergency Care Medicine, Pain Management (Prof. Dr. Dietrich Henzler, Dr. Klaas Eickmeyer, Dr. Juliane Krebs, Iris Rodenberg); Homburg, Homburg University Medical Centre, Department of Anaesthesiology, Intensive Care and Pain Medicine (Dr. Heinrich Groesdonk, Kathrin Meiers, Karen Salm, Prof. Dr. Thomas Volk); Ibbenbüren, Ibbenbüren General Hospital, Division of Thoracic Surgery and Lung Support (Prof. Dr. Stefan Fischer, Basam Redwan); Immenstadt, Kempten-Oberallgaeu Hospitals, Clinic for Pneumology, Thoracic Oncology, Sleep- and Respiratory Critical Care (Dr. Martin Schmölz, Dr. Kathrin Schumann-Stoiber, Simone Eberl); Ingolstadt, Klinikum Ingolstadt, Department of Anaesthesiology and Critical Care Medicine (Prof. Dr. Gunther Lenz, Thomas von Wernitz- Keibel, Monika Zackel); Jena, Jena University Hospital, Department of Anaesthesiology and Intensive Care Therapy (Dr. Frank Bloos, Dr. Petra Bloos, Anke Braune, Anja Haucke, Almut Noack, Steffi Kolanos, Heike Kuhnsch, Karina Knuhr-Kohlberg); Kassel, Klinikum Kassel, Department of Anaesthesiology (PD Dr. Markus Gehling); Kempten, Klinikum Kempten- Oberallgäu gGmbH, Department for Anaesthesia and Operative Intensive Care (Prof. Dr. Mathias Haller, Dr. Anne Sturm, Dr. Jannik Rossenbach); Kiel, University Medical Center Schleswig-Holstein, Campus Kiel, Department of Anaesthesiology and Intensive Care Medicine (Dr. Dirk Schädler, Stefanie D'Aria); Köln, Cologne-Merheim Hospital, Department of Pneumology and Critical Care Medicine (Prof. Dr. Christian Karagiannidis, Dr. Stephan Straßmann, Prof. Dr. Wolfram Windisch); Köln, University Hospital of Cologne, Department of Anaesthesiology and Intensive Care Medicine (Prof. Dr. Thorsten Annecke, PD Dr. Holger Herff); Langen, Asklepios Kliniken Langen-Seligenstadt, Department of Anaesthesiology and Intensive Care Medicine (Dr. Michael Schütz); Leipzig, University of Leipzig, Department of Anaesthesiology and Intensive Care Medicine (PD Dr. Sven Bercker, Hannah Reising, Mandy Dathe, Christian Schlegel); Ludwigsburg, Klinikum Ludwigsburg, Academic Teaching Hospital, University of Heidelberg, Department of Anaesthesiology (Katrin Lichy); Ludwigshafen, Klinikum Ludwigshafen, Department of Anaesthesiology and Intensive Care Medicine (Prof. Dr. Wolfgang Zink, Dr. Jana Kötteritzsch); Mainz, University Medical Centre Mainz, Department of Anaesthesiology (Dr. Marc Bodenstein, Susanne Mauff, Peter Straub); Magdeburg, Magdeburg University Medical Centre, Department of Anaesthesiology and

Intensive Care Medicine (Dr. Christof Strang, Florian Prätsch, Prof. Dr. Thomas Hachenberg); Mannheim, University Medical Centre Mannheim, Department of Anaesthesiology and Surgical Intensive Care Medicine (Dr. Thomas Kirschning, Dr. Thomas Friedrich, Dr. Dennis Mangold); Marburg, University Hospital, Department of Anaesthesiology (Dr. Christian Arndt, Tilo Koch); Mönchengladbach, Kliniken Maria-Hilf GmbH, Department of Cardiology (Dr. Hendrik Haake, Katrin Offermanns); München, Bogenhausen Hospital, Department of Anaesthesiology (Prof. Dr. Patrick Friederich, Dr. Florian Bingold); München, Klinikum Großhadern, Department of Anaesthesiology (Dr. Michael Irlbeck, Prof. Dr. Bernhard Zwissler); München, Klinikum Neuperlach, Städtisches Klinikum München GmbH, Department of Anaesthesiology, Critical Care and Pain Medicine (PD Dr. Ines Kaufmann); München, Klinikum rechts der Isar, Department for Anaesthesiology of the Technical University of Munich (Dr. Ralph Bogdanski, Dr. Barbara Kapfer, Dr. Markus Heim, PD Dr. Günther Edenharter); Münster, University Hospital Münster, Department for Anaesthesiology, Intensive Care Medicine and Pain Therapy, (Prof. Dr. Björn Ellger, Daniela Bause); Neumarkt, Kliniken des Landkreises Neumarkt i.d.OPf, Department for Anaesthesiology and Intensive Care Medicine (Dr. Götz Gerresheim); Nürnberg, General Hospital Nuremberg, Paracelsus Medical University, Department of Emergency Medicine and Intensive Care (Dr. Dorothea Muschner, Prof. Dr. Michael Christ, Arnim Geise); Osnabrück, Marienhospital Osnabrück, Department of Anaesthesiology (PD Dr. Martin Beiderlinden, Dr. Thorsten Heuter); Passau, Klinikum Passau, Department for Anaesthesiology (Dr. Alexander Wipfel); Regensburg, Caritas Krankenhaus St. Josef, Department for Anaesthesiology (Dr. Werner Kargl, Dr. Marion Harth, Dr. Christian Englmeier); Regensburg, Regensburg University Hospital, Department of Anaesthesiology, Operative Intensive Care (Prof. Dr. Thomas Bein, Dr. Sebastian Blecha, Dr. Kathrin Thomann-Hackner, Marius Zeder); Stuttgart, Katharinenhospital, Department of Anaesthesiology (Dr. Markus Stephan); Traunstein, Klinikum Traunstein, Department of Anaesthesiology (Dr. Martin Glaser); Tübingen, Tübingen University Hospital, Eberhard-Karls University Tübingen, Department of Anaesthesiology and Intensive Care Medicine (PD Dr. Helene Häberle); Ulm, Ulm University, Department of Anaesthesiology (Prof. Dr. Hendrik Bracht, Christian Heer, Theresa Mast); Würzburg, University of Würzburg, Department of Anaesthesia and Critical Care (PD Dr. Markus Kredel, PD Dr. Ralf Müllenbach).

Furthermore, we are grateful to previous members of the Regensburg DACAPO study team (medical documentation: Phillip Sebök, study physician: Kathrin Thomann-Hackner), to the members of the Advisory Board of the DACAPO-Study (Prof. Dr. Julika Loss, Prof. Dr.

Bernhard Graf, Prof. Dr. Michael Leitzmann, Prof. Dr. Michael Pfeifer, Regensburg, Germany), to our assisting students (Simon Bein, Vreni Brunnthaler, Carina Forster, Stefanie Hertling, Sophie Höhne, Carolin Schimmele, Elisa Valletta), and we are grateful to Monika Schoell for the linguistic revision of the manuscript.

# References

1. Matthay MA, Ware LB, Zimmerman GA. The acute respiratory distress syndrome. J Clin Invest. 2012; 122: 2731–2740. doi: 10.1172/JCI60331.
2. Karagiannidis C, Mostert C, Hentschker C, Voshaar T, Malzahn J, Schillinger G, et al. Case characteristics, resource use, and outcomes of 10 021 patients with COVID-19 admitted to 920 German hospitals. An observational study. The Lancet Respiratory Medicine. 2020; 8: 853–862. doi: 10.1016/S2213-2600(20)30316-7.
3. Botta M, Tsonas AM, Pillay J, Boers LS, Algera AG, Bos LDJ, et al. Ventilation management and clinical outcomes in invasively ventilated patients with COVID-19 (PRoVENT-COVID). A national, multicentre, observational cohort study. The Lancet Respiratory Medicine. 2021; 9: 139–148. doi: 10.1016/S2213-2600(20)30459-8.
4. Nelson BJ, Weinert CR, Bury CL, Marinelli WA, Gross CR. Intensive care unit drug use and subsequent quality of life in acute lung injury patients. Crit Care Med. 2000; 28: 3626–3630. doi: 10.1097/00003246-200011000-00013.
5. Tanaka LMS, Azevedo LCP, Park M, Schettino G, Nassar AP, Rea-Neto A, et al. Early sedation and clinical outcomes of mechanically ventilated patients: a prospective multicenter cohort study. Crit Care. 2014; 18: R156. doi: 10.1186/cc13995.
6. Bienvenu OJ, Friedman LA, Colantuoni E, Dinglas VD, Sepulveda KA, Mendez-Tellez P, et al. Psychiatric symptoms after acute respiratory distress syndrome: a 5-year longitudinal study. Intensive Care Med. 2018; 44: 38–47. doi: 10.1007/s00134-017- 5009-4.
7. Bein T, Weber-Carstens S, Apfelbacher C. Long-term outcome after the acute respiratory distress syndrome: different from general critical illness. Curr Opin Crit Care. 2018; 24: 35–40. doi: 10.1097/MCC.0000000000000476.
8. Latronico N, Peli E, Calza S, Rodella F, Novelli MP, Cella A, et al. Physical, cognitive and mental health outcomes in 1-year survivors of COVID-19-associated ARDS. Thorax. 2021. doi: 10.1136/thoraxjnl-2021-218064.
9. Aranda J, Oriol I, Martín M, Feria L, Vázquez N, Rhyman N, et al. Long-term impact of COVID-19 associated acute respiratory distress syndrome. J Infect. 2021.

doi: 10.1016/j.jinf.2021.08.018.

10. Brandstetter S, Dodoo-Schittko F, Blecha S, Sebök P, Thomann-Hackner K, Quintel M, et al. Influence of quality of care and individual patient characteristics on quality of life and return to work in survivors of the acute respiratory distress syndrome. Protocol for a prospective, observational, multi-centre patient cohort study (DACAPO). BMC Health Serv Res. 2015; 15: 563. doi: 10.1186/s12913-015-1232-2.
11. Dodoo-Schittko F, Brandstetter S, Brandl M, Blecha S, Quintel M, Weber-Carstens S, et al. German-wide prospective DACAPO cohort of survivors of the acute respiratory distress syndrome (ARDS). A cohort profile. BMJ Open. 2018; 8: e019342.

doi: 10.1136/bmjopen-2017-019342.

12. Dodoo-Schittko F, Brandstetter S, Brandl M, Blecha S, Quintel M, Weber-Carstens S, et al. Characteristics and provision of care of patients with the acute respiratory distress syndrome. Descriptive findings from the DACAPO cohort baseline and comparison with international findings. J Thorac Dis. 2017; 9: 818–830. doi: 10.21037/jtd.2017.03.120.
13. Ranieri VM, Rubenfeld GD, Thompson BT, Ferguson ND, Caldwell E, Fan E, et al. Acute respiratory distress syndrome. The Berlin Definition. JAMA. 2012; 307: 2526– 2533. doi: 10.1001/jama.2012.5669.
14. Blecha S, Brandl M, Zeman F, Dodoo-Schittko F, Brandstetter S, Karagiannidis C, et al. Tracheostomy in patients with acute respiratory distress syndrome is not related to quality of life, symptoms of psychiatric disorders or return-to-work. The prospective DACAPO cohort study. Ann. Intensive Care. 2020; 10: 2731. doi: 10.1186/s13613-020- 00671-x.
15. Kroenke K, Spitzer RL, Williams JB. The PHQ-9. Validity of a brief depression severity measure. J Gen Intern Med. 2001; 16: 606–613. doi: 10.1046/j.1525- 1497.2001.016009606.x.
16. Parker AM, Sricharoenchai T, Raparla S, Schneck KW, Bienvenu OJ, Needham DM. Posttraumatic stress disorder in critical illness survivors. A metaanalysis. Crit Care Med. 2015; 43: 1121–1129. doi: 10.1097/CCM.0000000000000882.
17. Bullinger M. German translation and psychometric testing of the SF-36 Health Survey. Preliminary results from the IQOLA project. Social Science & Medicine. 1995; 41: 1359–1366. doi: 10.1016/0277-9536(95)00115-n.
18. Stieglitz R-D. Bullinger, M. & Kirchberger, I. (1998). SF-36. Fragebogen zum Gesundheitszustand. Göttingen. Hogrefe. Preis DM 298,-. Zeitschrift für Klinische Psychologie und Psychotherapie. 1999; 28: 143–145. doi: 10.1026//0084-5345.28.2.143.
19. Piriyapatsom A, Bittner EA, Hines J, Schmidt UH. Sedation and paralysis. Respir Care. 2013; 58: 1024–1037. doi: 10.4187/respcare.02232.
20. Strøm T, Martinussen T, Toft P. A protocol of no sedation for critically ill patients receiving mechanical ventilation. A randomised trial. The Lancet. 2010; 375: 475–480. doi: 10.1016/S0140-6736(09)62072-9.
21. Ren X, Ai Y, Zhang L, Zhao C, Li L, Ma X. Sedation and analgesia requirements during venovenous extracorporeal membrane oxygenation in acute respiratory distress syndrome patients. Perfusion. 2021: 2676591211052160. doi: 10.1177/02676591211052160.
22. Stevenson JE, Colantuoni E, Bienvenu OJ, Sricharoenchai T, Wozniak A, Shanholtz C, et al. General anxiety symptoms after acute lung injury: predictors and correlates. J Psychosom Res. 2013; 75: 287–293. doi: 10.1016/j.jpsychores.2013.06.002.
23. Herridge MS, Tansey CM, Matté A, Tomlinson G, Diaz-Granados N, Cooper A, et al. Functional disability 5 years after acute respiratory distress syndrome. N Engl J Med. 2011; 364: 1293–1304. doi: 10.1056/NEJMoa1011802.
24. Mion G, Villevieille T. Ketamine pharmacology. An update (pharmacodynamics and molecular aspects, recent findings). CNS Neurosci Ther. 2013; 19: 370–380.

doi: 10.1111/cns.12099.

25. Fond G, Loundou A, Rabu C, Macgregor A, Lançon C, Brittner M, et al. Ketamine administration in depressive disorders. A systematic review and meta-analysis. Psychopharmacology (Berl). 2014; 231: 3663–3676. doi: 10.1007/s00213-014-3664-5.
26. Serafini G, Howland RH, Rovedi F, Girardi P, Amore M. The role of ketamine in treatment-resistant depression. A systematic review. Curr Neuropharmacol. 2014; 12: 444–461. doi: 10.2174/1570159X12666140619204251.
27. Köhler S, Betzler F. Ketamin - Eine neue Option in der Behandlung der therapieresistenten Depression. Fortschr Neurol Psychiatr. 2015; 83: 91–97. doi: 10.1055/s-0034-1398967.
28. Wu TT, Ko S, Kooken R, van den Boogaard M, Devlin JW. Exploring Ketamine Analgosedation Use and Its Effect on Incident Delirium in Critically Ill Adults. Crit Care Explor. 2021; 3: e0544. doi: 10.1097/CCE.0000000000000544.
29. Jaeger M, Attridge RL, Neff LA, Gutierrez GC. Safety and Effectiveness of Sedation With Adjunctive Ketamine Versus Nonketamine Sedation in the Medical Intensive Care Unit. J Pharm Pract. 2021; 34: 850–856. doi: 10.1177/0897190020925932.
30. Martin J, Franck M, Fischer M, Spies C. Sedation and analgesia in German intensive care units. How is it done in reality? Results of a patient-based survey of analgesia and sedation. Intensive Care Med. 2006; 32: 1137–1142. doi: 10.1007/s00134-006-0214-6.
31. Patel RP, Gambrell M, Speroff T, Scott TA, Pun BT, Okahashi J, et al. Delirium and sedation in the intensive care unit. Survey of behaviors and attitudes of 1384 healthcare professionals. Crit Care Med. 2009; 37: 825–832.

doi: 10.1097/CCM.0b013e31819b8608.

32. Casault C, Soo A, Lee CH, Couillard P, Niven D, Stelfox T, et al. Sedation strategy and ICU delirium. A multicentre, population-based propensity score-matched cohort study. BMJ Open. 2021; 11: e045087. doi: 10.1136/bmjopen-2020-045087.
33. Fraser GL, Devlin JW, Worby CP, Alhazzani W, Barr J, Dasta JF, et al. Benzodiazepine versus nonbenzodiazepine-based sedation for mechanically ventilated, critically ill adults. A systematic review and meta-analysis of randomized trials. Crit Care Med. 2013; 41: S30-8. doi: 10.1097/CCM.0b013e3182a16898.
34. Ely EW, Shintani A, Truman B, Speroff T, Gordon SM, Harrell FE, et al. Delirium as a predictor of mortality in mechanically ventilated patients in the intensive care unit. JAMA. 2004; 291: 1753–1762. doi: 10.1001/jama.291.14.1753.
35. Zaal IJ, Devlin JW, Peelen LM, Slooter AJC. A systematic review of risk factors for delirium in the ICU. Crit Care Med. 2015; 43: 40–47.

doi: 10.1097/CCM.0000000000000625.

# Supporting information

**S1 Table:** Multiple linear regression models of the influence of cumulative analgosedation (without ketamine and midazolam) on psychiatric symptoms in ARDS survivors 3, 6 and 12 months after ICU discharge**.**

S1 Table legend: *p <0.05; **per 1000 mg; ^#^further adjusted for age, sex, SAPS, days on ICU; B: regression coefficient, 95%-CI: 95% confidence interval, PHQ-9: Patient Health Questionnaire-9; PTSS-14: Post-Traumatic Stress Syndrome 14-Questions Inventory; ^a^symptoms of psychiatric disorders (depression and PTSD) were diagnosed according to the results of patient self-reported questionnaires

# Figures legends

**Fig. 1:** Flow chart of patients with ARDS throughout the study

**Fig. 2:** Relationship between cumulative dose of ketamine and psychiatric symptoms in ARDS survivors 3, 6 and 12 months after ICU discharge

**Fig. 3:** Relationship between cumulative dose of midazolam and psychiatric symptoms in ARDS survivors 3, 6 and 12 months after ICU discharge

# Tables legends

**Tab. 1:** Patient characteristics of ARDS survivors (n=134)

**Tab. 2:** Total cumulative dose and duration of sedation and analgesia in ARDS survivors (n=134)

**Tab. 3:** HRQoL and symptoms of psychiatric disorders in ARDS survivors after 3, 6 and 12 months (n=84)

**Tab. 4:** Multiple linear regression models of the influence of cumulative ketamine doses on psychiatric symptoms in ARDS survivors 3, 6 and 12 months after ICU discharge

Figure 1	[Click here to access/download;Figure;Figure 1-061221.jpg](https://www.editorialmanager.com/pone/download.aspx?id=31645538&guid=c81896a9-e6fe-4e1b-ac92-4edbcc2b9411&scheme=1)

[.:!](https://www.editorialmanager.com/pone/download.aspx?id=31645538&guid=c81896a9-e6fe-4e1b-ac92-4edbcc2b9411&scheme=1)

I!

ARDS :S lf li'YWCf':S

n.:ti,n

1 i,sfl

D-

me1cnat11L:' fu:iT1I 'ICU

I

fi

)'Ii IJ.l'QII! llU!i due

l	I:*u;*IUl\ifroMI a,xlr,E::5$ [n,a.S&.]

1Jn(Jblc Ii) p1, 1."i;:ip.ni.ci ('1

**:a	rlm:vtl'•JH,J-,..nn.!!l't r_ l11·r.19,** en=a,

,

poR!:i [(1• 1-4

I

"')I!

i!looi'.ifl **and**

oll Mdallill\ Md

.(JE<la, IJOl'I

Ro omlm1ts w 1h doeumontedl s,s,	1;il.VQS dm 'li lflg ICU traaiffl9J'[it n==134

R,ospa:ndents aflo:r ·1^1^2 months.

n Sl:6

Wrllt:om inf-ormod consent at ICU

... 1225

Figure 2	[Click here to access/download;Figure;Figure 2-040322.jpg](https://www.editorialmanager.com/pone/download.aspx?id=31645539&guid=b1072d2d-2624-4c4c-81ef-ab63c3fc62fa&scheme=1)

**l mJJ**■**lh	11**

.,

..

., .,.

II

## ..

,.

#### ..

,.

,■

**C' ,**

&

f

.1	..

## ..

.I •...

,

, •

#### . ,

**!I!**

.,

;:!;

,.

## ..

.,

,.,.

J	!111!11

,., **,ji**

•·

·•·	,.

Figure 3	[Click here to access/download;Figure;Figure 3-040322.jpg](https://www.editorialmanager.com/pone/download.aspx?id=31645540&guid=e74b6362-7c53-4055-9cfd-01318730c37e&scheme=1)

**l mJJ**■**lh	11**

;

ii

·•·

"

" '"

·•,.

" ,.

....

;--

..

....,

,,..

,,

..

"'

**ll**

**1l l 1 lllil l**

**ll** ,.

-

.1.iiii1

..

"

,.

..

,

•·

,l,

,I

II

..

"'

,.

,■

•

iii

..,.	.,	..

..

"'

f

**t**

·•

,,,

***J'***

I , ll

I

***l4***

'"

_,._	,.

**1 1	I •**

1 !! '

,.

"

**lil**.**lll**.**!**.. **rl**

"

## ..

. "

## ..

I!!!	I■

**ii**	..

..

,., Iii

"

Supporting Information

[Click here to access/download](https://www.editorialmanager.com/pone/download.aspx?id=31645577&guid=bd3e3f8c-ed8d-49b2-b21e-7ecbe0068973&scheme=1)

[**Supporting Information** S1 Table 030822.pdf](https://www.editorialmanager.com/pone/download.aspx?id=31645577&guid=bd3e3f8c-ed8d-49b2-b21e-7ecbe0068973&scheme=1)

Revised Manuscript with Track Changes

# Association of analgosedation with psychiatric symptoms and health-related quality of life in ARDS survivors: Post hoc analyses of the DACAPO study

Sebastian Blecha^1*^, [Sebastian.Blecha@ukr.de](mailto:Sebastian.Blecha@ukr.de) Florian Zeman^2^, [Florian.Zeman@ukr.de](mailto:Florian.Zeman@ukr.de)

Magdalena Rohr^3^, [Magdalena.Rohr@klinik.uni-regensburg.de](mailto:Magdalena.Rohr@klinik.uni-regensburg.de)

Frank Dodoo-Schittko^3,#a^, [Frank.Dodoo-Schittko@klinik.uni-regensburg.de](mailto:Frank.Dodoo-Schittko@klinik.uni-regensburg.de) Susanne Brandstetter^3,#b^, [Susanne.Brandstetter@klinik.uni-regensburg.de](mailto:Susanne.Brandstetter@klinik.uni-regensburg.de) Christian Karagiannidis^4^, [karagiannidisc@kliniken-koeln.de](mailto:karagiannidisc@kliniken-koeln.de)

Christian Apfelbacher^3,#a^, [Christian.Apfelbacher@med.ovgu.de](mailto:Christian.Apfelbacher@med.ovgu.de) Thomas Bein^5^, [Thomas.Bein@ukr.de](mailto:Thomas.Bein@ukr.de)

for the DACAPO study group

1 Department of Anaesthesiology, University Medical Centre Regensburg, Regensburg, Germany
2 Centre of Clinical Studies, University Medical Centre Regensburg, Regensburg, Germany
3 Medical Sociology, Institute of Epidemiology and Preventive Medicine, University of Regensburg, Regensburg, Germany
4 Department of Pneumology and Critical Care Medicine, Cologne-Merheim Hospital, ARDS and ECMO Centre, Kliniken der Stadt Köln gGmbH, Witten/Herdecke University Hospital, Cologne, Germany
5 Faculty of Medicine, University of Regensburg, Regensburg, Germany

#a	Current Address: Institute of Social Medicine and Health Systems Research, Otto-von- Guericke-University Magdeburg, Magdeburg, Germany

#b	Current Address: University Children‘s Hospital Regensburg (KUNO), University of Regensburg, Regensburg, Germany

*Corresponding author:

Email: [Sebastian.Blecha@ukr.de](mailto:Sebastian.Blecha@ukr.de)

Trial registration:

Clinicaltrials.gov: NCT02637011 (Registered 15 December 2015, retrospectively registered)

**Word count:** 2436

# Abstract

**Background:** The acute respiratory distress syndrome (ARDS) is a life-threatening condition with the risk of developing hypoxia and thus requires for invasive mechanical ventilation a long-term analgosedation. Yet, prolonged analgosedation may be a reason for declining health-related quality of life (HRQoL) and the development of psychiatric disorders.

**Methods:** We used data from the prospective observational nation‑ wide ARDS study across Germany (DACAPO) to investigate the influence of sedation and analgesia on HRQoL and the risk of psychiatric symptoms in ARDS survivors 3, 6 and 12 months after their discharge from the intensive care unit (ICU). HRQoL was measured with the Physical and Mental Component Scale of the Short‑ Form 12 Questionnaire (PCS‑ 12, MCS‑ 12). The prevalence of psychiatric symptoms (depression and post‑ traumatic stress disorder [PTSD]) was assessed using the Patient Health Questionnaire‑ 9 and the Post‑ Traumatic Stress Syndrome‑ 14. The associations of analgosedation with HRQoL and psychiatric symptoms were investigated by means of multivariable linear regression models.

**Results:** The data of 134 ARDS survivors (median age [IQR]: 55 [44–64], 67% men) did not show any significant association between analgosedation and physical or mental HRQoL up to 1 year after ICU discharge. Multivariable linear regression analysis (B [95%‑ CI]) yielded a significant association between symptoms of psychiatric disorders and increased cumulative doses of ketamine up to 6 months after ICU discharge (after 3 months: depression: 0.15 [0.05, 0.25]; after 6 months: depression: 0.13 [0.03, 0.24] and PTSD: 0.42 [0.04, 0.80)]).

**Conclusions:** Up to 1 year after ICU discharge, analgosedation did not influence HRQoL of ARDS survivors. Prolonged administration of ketamine during ICU treatment, however, was positively associated with the risk of psychiatric symptoms. The administration of ketamine to ICU patients with ARDS should be with caution.

**Keywords:** Acute respiratory distress syndrome, analgosedation, depression, posttraumatic stress disorder, HRQoL

# Background

The acute respiratory distress syndrome (ARDS) is a life-threatening condition, which is characterised by either direct or indirect damage to the lung parenchyma, often resulting in critical hypoxemia or hypercapnia, or both [1]. In the initial phase of ARDS, patients require long-term analgosedation for invasive mechanical ventilation. In the current context, patients with severe COVID-19-associated ARDS are usually ventilated for a mean duration of 13 to 16 days [2,3]. Prolonged sedation during intensive care therapy may be a potential risk factor for the development of psychiatric disorders and delirium [4,5].

Additionally, ARDS survivors have a substantial risk of developing anxiety, depression and symptoms of posttraumatic stress disorder (PTSD) as well as of decreased health-related quality of life (HRQoL)[6,7]. A recent study of 114 COVID-19-associated ARDS survivors in Italy showed symptoms of depression in 9% of patients, of anxiety in 10% and of PTSD in 4% [8]. A study of 113 COVID-19-associated ARDS survivors in Spain showed that over 90% of patients had developed one or more symptoms of a mental disorder [9].

In the current study, we assessed the type and dosage of sedation and analgesia administered during intensive care treatment and the influence of analgosedation on HRQOL and psychiatric disorders up to 1 year after ARDS survival. We hypothesised that analgesics and sedation drugs and their administered doses during ICU treatment increased the risk of psychiatric symptoms and reduced HRQoL of long-term survivors of ARDS.

# Methods

### Study design

The extent and duration of analgosedation and co-medications of ARDS survivors was recorded in the context of a large prospective nation-wide cohort study across Germany

(DACAPO study, ClinicalTrials.gov Identifier: NCT02637011) [10]. The DACAPO study was focused on the influence of the quality of care and individual patient characteristics on

HRQoL and the time point of returning to work of ARDS survivors. Ethical approval was

obtained from the ethics committee of the University of Regensburg (file number: 13–101-

0262) and additionally from the ethics committees overseeing the respective study sites [11]. The baseline characteristics and profile of the cohort have been described in more detail elsewhere [12]. ARDS survivors were asked to complete comprehensive self-report questionnaires 3, 6 and 12 months after their discharge from the intensive care unit (ICU).

### Sample

**Fig. 1** gives an overview of the sample size at different time points of the study. Written

informed consent was obtained from 1,225 patients with ARDS, who had been treated at one of 61 ICUs across Germany between September 2014 and April 2016. The study included adults with the diagnosis of ARDS (according to the Berlin definition [13]). Patients or their caregivers or legal guardians were approached during the patients’ ICU stay and asked to provide written informed consent. In cases in which caregivers or legal guardians consented to a patient’s participation in the study, the patient had to confirm this preliminary consent at discharge from the ICU.

Out of 877 ICU survivors, 396 (45.2%) had returned the questionnaire at 12 months. The most frequent reason for dropping out of the study was death after discharge from the ICU

(N=161). Other reasons included the inability to complete the questionnaire (insufficient

knowledge of German or incapability due to morbidity), absence of a person who could provide proxy reports, withdrawal of consent or an invalid address. Furthermore, the doses and duration of analgosedation could not be assessed in 262 ARDS survivors.

### Data collection and measuring instruments

Characteristics of patient disease and treatment as well as information on ICU discharge were reported by study physicians or physicians from the participating ICUs using the electronic data capture system OpenClinica (OpenClinica, LLC; https:/[/www.openclinica.com/).](http://www.openclinica.com/)) The

type of analgesia and sedation, duration and cumulative doses of drugs were extracted and

transmitted from the patient data management system of nine recruiting study centres (8

University hospitals and one hospital for basic and standard care). Information on HRQoL and psychiatric disorders was assessed by means of self-report questionnaires at 3, 6 and 12 months after discharge from the ICU. ARDS survivors completed the Short Form-12 self- report questionnaire (SF-12) as a measure of HRQoL and questionnaires on depressive symptoms and post-traumatic stress disorder (PTSD). The Patient Health Questionnaire-9 [PHQ-9] was used to detect symptoms of depression and the Post-Traumatic Stress Syndrome

14-Questions Inventory [PTSS-14] to detect PTSD [14]. For the screening tools, the cut-off

values for being at risk of depression were defined as PHQ-9 ≥5 and for PTSD as PTSS-14

≥45 [15,16]. The published scoring algorithm of SF-12 resulted in the Physical Component Summary (PCS-12) and the Mental Component Summary (MCS-12) scores [2]. Scores range from 0 to 100 (higher values indicate better HRQoL); a score of 50 represents the mean value for the general population (German norm values [17,18]).

### Statistical analyses

All patients of the prospective DACAPO study with data on analgosedation were used for this

supplementary analysis. Thus, no more than n=134 patients were available. Nevertheless, this

sample is sufficient for the first exploratory analyses regarding our additional questions. As a

rule of thumb, a minimum of 10-12 cases is needed per variable for a linear regression model.

In our multiple linear regression model with eight independent variables, we needed at least

80 cases for the first exploratory analyses. Data are shown as mean ± SD or median (IQR) for continuous variables and as absolute and relative frequencies for categorical variables. To assess which analgosedation has a potential impact on HRQoL and the risk of psychiatric symptoms after 3, 6 and 12 months, bivariate models (including type of analgosedation yes/no plus cumulative doses) were calculated for each type of analgosedation and each endpoint. For each endpoint, all significant results were included in a multiple linear regression model,

which was further adjusted for the known risk factors age, sex, Simplified Acute Physiology

Score II (SAPS II) and length of the ICU stay. The regression coefficient B for the linear regression models was calculated as effect estimate accompanied by the corresponding 95%- confidence interval (95%-CI). A p-value <0.05 was considered significant. Due to the exploratory nature of this study, no adjustments for multiple comparisons were used. All analyses were performed using the software R (Version 3.5.1, [www.r-project.org](http://www.r-project.org/)).

# Results

Out of 396 responding ARDS survivors, 134 patients were analysed 12 months after their ICU discharge (**Figure 1**). The patient characteristics are shown in **Table 1**. Median age at ICU admission was 55 years, two-thirds of the 12-month respondents were men and median length of the ICU stay was 23.5 days. The majority of patients had moderate or severe ARDS (82.1%). Tracheostomy was performed in 46% of ARDS survivors after a median time of 11 days (IQR: 7–16).

**Table 1:** Patient characteristics of ARDS survivors with documented analgosedation (n=134)

| **Patient characteristics** |  | **Missing** (n) |
| --- | --- | --- |
| **Sex** | | |
| Men, n (%) | 90 (67) | 0 |
| Women, n (%) | 44 (33) |  |
| **Age,** years, median (IQR) | 55 (44-64) | 0 |
| **BMI (kg/m²)**, median (IQR) | 26 (24-30) | 0 |
| **Severity of ARDSa** | | |
| Mild, n (%) | 24 (17.9) | 0 |
| Moderate, n (%) | 75 (56.0) |  |
| Severe, n (%) | 35 (26.1) |  |
| **SAPSb,** median (IQR) | 38 (30-46) | 5 |
| **SOFA scorea,** median (IQR) | 8 (6-11) | 9 |
| **Duration of ICU stay,**  days, median (IQR) | 23.5 (15-35) | 0 |
| **Tracheotomy during ICU stay** | | |
| Yes, n (%) | 61 (46) | 1 |
| No, n (%) | 72 (54) |  |

Notes: ARDS: acute respiratory distress syndrome, BMI: body mass index, ICU: intensive care unit, SAPS: Simplified Acute Physiology Score, SOFA: Sequential Organ Failure Assessment; ^a^measured at the time of ARDS diagnosis, ^b^measured 24 hours after ICU admission

The drugs used for analgesia and sedation during ICU treatment in ARDS survivors are

shown in **Table 2**. In the following, the number of sedative and analgesic agents that patients

received during ICU treatment is listed (based on Table 2): 12 patients (8.9%) got a sixfold,

28 patients (20.9%) fivefold, 38 patients (28.4%) a fourfold, 34 patients (25.4%) a threefold,

19 patients (14.2%) twofold and only 3 patients (2.2%) single analgosedation. The most common type of analgosedation was the combination of intravenously administered propofol

and sufentanil. Sedation was often deepened with clonidine (73.1%) and/or midazolam

(59.0%). Ketamine or esketamine was administered in 32.1% of patients. Thirty-two of 39

patients (82.1%) with ketamine application and all patients with esketamine additionally

received midazolam. Inhalative sedation with isoflurane administered via the AnaConDa® system was used in only 6% of patients.

**Table 2:** Total cumulative dose and duration of sedation and analgesia in ARDS survivors (N=134)

| **Sedation and analgesic agents** | **n (**%**)** | **Total cumulative dose**  mg, median (IQR) | **Duration of sedation and analgesia**  days, median (IQR) |
| --- | --- | --- | --- |
| **Propofol** | 129 (96.3) | 21355 (11786–39177) | 7 (4–10) |
| **Sufentanil** | 124 (92.5) | 5121 (23–18386) | 12 (7–20) |
| **Midazolam** | 79 (59.0) | 1084 (295–4100) | 6 (3–14) |
| **Ketamine** | 39 (29.1) | 8830 (2247–31034) | 3 (1–7) |
| **Esketamine** | 4 (3.0) | 5225 (164–10260) | 5 (2–8) |
| **Clonidine** | 98 (73.1) | 7 (3–20) | 5 (2–10) |
| **Dexmedetomidine** | 20 (14.9) | 0.21 (0.03–2.62) | 4 (2–8) |
| **Isoflurane** | 8 (6.0) | - | 8 (4–15) |

HRQoL and symptoms of psychiatric disorders of ARDS survivors 3, 6 and 12 months after ICU discharge are shown in **Table 3**. From 134 patients, for whom medication was documented during ICU therapy, the values of SF-12, PHQ-9, and PTSS-14 of 84 ARDS survivors could be compared. According to SF-12 measurement, ARDS survivors had lower HRQoL (<50) across all follow-up time points compared to the general population norm [18]. Additionally, the mean increased PHQ-9 values show symptoms of depression across all follow-up time points. 20.2% of patients reported symptoms of PTSD (cut-off scores for symptoms of PTSD: ≥45) and 54.2 % symptoms of depression (PHQ-9 score ≥5) at the 1-year follow-up, respectively.

**Table 3**: HRQoL and symptoms of psychiatric disorders in ARDS survivors after 3, 6 and 12 months (N=84)

|  |  | | **Missing** (n) |
| --- | --- | --- | --- |
| **3 months after ICU discharge** | | | |
| **PCS-12a,** mean (±SD); ^#^ n (%) | 36.6 (±9.5) ^#^ | ^#^ 64 (87.7) | 11 |
| **MCS-12a,** mean (±SD); ^#^ n (%) | 43.7 (±12.1) ^#^ | ^#^ 46 (63.0) | 11 |
| **PHQ-9a,** mean (±SD); *n (%) | 5.6 (±4.7)* | * 38 (45.8) | 1 |
| **PTSS-14a,** mean (±SD); *n (%) | 29.1 (±13.5) | * 12 (14.5) | 1 |
| **6 months after ICU discharge** | | | |
| **PCS-12a,** mean (±SD); ^#^ n (%) | 40.2 (±11.8) ^#^ | ^#^ 47 (75.8) | 22 |
| **MCS-12a,** mean (±SD); ^#^ n (%) | 42.1 (±14.4) ^#^ | ^#^ 40 (64.5) | 22 |
| **PHQ-9a,** mean (±SD); *n (%) | 6.1 (±5.1)* | * 45 (54.2) | 1 |
| **PTSS-14a,** mean (±SD); *n (%) | 32.2 (±17.7) | * 16 (20.0) | 4 |
| **12 months after ICU discharge** | | | |
| **PCS-12a,** mean (±SD); ^#^ n (%) | 42.7 (±11.7) ^#^ | ^#^ 46 (63.9) | 12 |
| **MCS-12a,** mean (±SD); ^#^ n (%) | 45.6 (±13.9) ^#^ | ^#^ 38 (52.8) | 12 |
| **PHQ-9a,** mean (±SD); *n (%) | 5.8 (±5.2)* | * 45 (54.2) | 1 |
| **PTSS-14a,** mean (±SD); *n (%) | 31.5 (±16.8) | * 17 (20.2) | 0 |

Notes: MCS-12: mental component scale of short-form 12 questionnaire; PCS-12: physical component scale of short-form 12 questionnaire; PTSD: post-traumatic stress disorder, PHQ- 9: Patient Health Questionnaire-9, PTSS-14: Post-Traumatic Stress Syndrome 14-Questions Inventory, ^a^health-related quality of life and symptoms of psychiatric disorders (depression, PTSD) were assessed according to the results of patient self-reported questionnaires; # PCS- 12<50 and MCS-12<50 represents worse HRQoL compared to that of the general (German) population; *cut-off values for being at risk of depression and PTSD: PHQ-9-score ≥5 and PTSS-14-score ≥45

Analysing the impact of analgosedation of all given drugs (propofol, midazolam, sufentanil, [es-]ketamine, clonidine, dexmedetomidine and isoflurane) on HRQoL and symptoms of psychiatric disorders during the follow-up time points, only the cumulative doses of ketamine and midazolam showed a significant linear relationship to PHQ-9 and PTSS-14 in the bivariable linear regression models. The relationship of ketamine to PHQ-9 and PTSS-14 scores is shown in **Fig. 2** and that of midazolam to PHQ-9 and PTSS-14 scores in **Fig. 3**. HRQoL (PCS-12/MCS-12) was not affected by analgesia and sedation at any follow-up time point.

Multivariable linear regression analysis only established the cumulative dose of ketamine as a significant predictor for depression and PTSD (**Table 4 and S1**). Higher doses of ketamine increased the risk of psychiatric symptoms in ARDS survivors up to 6 months after ICU

discharge. The number of drugs for analgosedation showed no influence on the psychiatric

outcome in the multivariable linear regression analysis.

**Table 4:** Multiple linear regression models of the influence of cumulative ketamine doses on psychiatric symptoms in ARDS survivors 3, 6 and 12 months after ICU discharge

|  | **PHQ-9a** | | **PTSS-14a** | |
| --- | --- | --- | --- | --- |
| **Independent variables#** | **B (95%-CI)** | **P-value** | **B (95%-CI)** | **P-value** |
|  | **3 months after ICU discharge** | | | |
| Ketamine | –1.5 (-4.3, 1.3) | 0.297 | –0.12 (–7.74, 7.50) | 0.975 |
| Cumulative ketamine dose** | 0.15 (0.05, 0.25) | **0.004*** | 0.24 (–0.05, 0.53) | 0.102 |
| Midazolam | 0.60 (–1.83, 3.02) | 0.625 | 2.53 (–4.27, 9.33) | 0.461 |
| Cumulative midazolam dose** | –0.09 (–0.61, 0.44) | 0.742 | 0.96 (–0.69, 2.61) | 0.248 |
|  | **6 months after ICU discharge** | | | |
| Ketamine | –2.8 (–5.8, 0.19) | 0.066 | –9.94 (–20.86, 0.99) | 0.074 |
| Cumulative ketamine dose** | 0.13 (0.03, 0.24) | **0.014*** | 0.42 (0.04, 0.80) | **0.029*** |
| Midazolam | –2.0 (–4.7, 0.67) | 0.138 | –2.65 (–12.31, 7.02) | 0.587 |
| Cumulative midazolam dose** | 0.29 (–0.20, 0.78) | 0.241 | 0.63 (–1.14, 2.40) | 0.479 |

|  | **12 months after ICU discharge** | | | |
| --- | --- | --- | --- | --- |
| Ketamine | –2.68 (–5.97, 0.62) | 0.110 | –8.58 (–19.53, 2.38) | 0.123 |
| Cumulative ketamine dose** | 0.03 (–0.08, 0.15) | 0.572 | 0.15 (–0.24, 0.53) | 0.453 |
| Midazolam | 0.04 (–2.80, 2.88) | 0.978 | –0.62 (–10.00, 8.75) | 0.895 |
| Cumulative midazolam dose** | 0.09 (–0.47, 0.64) | 0.761 | 0.46 (–1.36, 2.29) | 0.616 |

Notes: *p <0.05; **per 1000 mg; ^#^further adjusted for age, sex, SAPS, days on ICU; B: regression coefficient, 95%-CI: 95% confidence interval, PHQ-9: Patient Health Questionnaire-9; PTSS-14: Post-Traumatic Stress Syndrome 14-Questions Inventory; ^a^symptoms of psychiatric disorders (depression and PTSD) were diagnosed according to the results of patient self-reported questionnaires

# Discussion

This study analysed the influence of sedation and analgesia on HRQoL and the prevalence of psychiatric symptoms in 134 ARDS survivors up to 1 year after ICU discharge. The main findings were: 1) Increased cumulative ketamine doses for analgosedation during ICU treatment were significantly associated with a higher prevalence of psychiatric symptoms 3 and 6 months after ICU discharge and 2) Analgosedation was not significantly associated with physically (PCS-12) or mentally impaired quality of life (MCS-12).

##### Analgosedation during ICU treatment

At the onset of ARDS, deep analgosedation is often needed. After initial stabilisation during the recovery phase, a protocol-based sedation regimen emphasising light or no sedation, daily spontaneous awakening and breathing periods as well as physiotherapy may improve short- term outcome and decrease long-term complications including the risk of cognitive decline and psychiatric symptoms [19,20].

In our study, the most common type of analgosedation during ICU treatment was the combination of intravenously administered propofol and sufentanil, often supplemented or replaced with midazolam, clonidine or dexmedetomidine. Ketamine was administered in

nearly one third of the patients. In a recent study by Ren and colleagues, all 42 patients with ARDS who received venovenous extracorporeal membrane oxygenation were given midazolam combined with sufentanil or remifentanil; in 69%, analgosedation was supplemented with propofol and in 25% with dexmedetomidine [21].

##### HRQoL and psychiatric symptoms in ARDS survivors

The literature for analysing the association between analgosedation and the development of

psychiatric symptoms in ARDS survivors is very scarce. Stevenson and colleagues found that

38% of 152 ARDS survivors had a positive screening test for general anxiety 3 months after

ICU discharge, and pre-ICU body mass index and psychiatric comorbidity were negatively

associated with the development of general anxiety, but without any association of daily

administered analgesics, sedatives and steroids with general anxiety symptoms [22]. Bienvenu et al. showed in a 5-year follow-up study that generally 32% of ARDS survivors had prolonged symptoms of depression, 38% of anxiety and 23% of PTSD [6]. Furthermore, the Physical and Mental Component Score often shows decreased HRQoL in ARDS survivors in the long-term follow up [23]. In our study, ARDS survivors had lower HRQoL measured with the PSC-12 and MCS-12 at each follow-up time point compared to the score validated from the general population [17]. Except for ketamine, analgosedation had no impact on HRQoL or the development of psychiatric symptoms. In this post hoc-analysis, 20.2% of patients had symptoms of PTSD and 54.2 % symptoms of depression at the 1-year follow-up.

##### Indications and risks of ketamine and midazolam administration

Ketamine is an established sedative with a unique mechanism of action and side effect profile. The primary mechanism of action is antagonism of the N-methyl-D-aspartate receptor, which appears to be responsible for the amnestic and analgesic effects. In addition, ketamine stimulates noradrenergic neurons and inhibits catecholamine uptake, which may preserve cardiovascular stability. Ketamine also enhances the descending inhibiting serotoninergic

pathway and exerts anti-depressive effects [24]. This is the reason why ketamine is commonly used in patients with major depressive disorders, bipolar depression and treatment-resistant depression [25–27].

A recent prospective study including more than 1000 critically ill surgical and medical patients requiring analgosedation suggested that ketamine may also increase the risk of incident delirium [28]. However, another study including 172 ICU patients showed adjunctive continuous infusion with ketamine to be safe and effective for sedation without any increased risk of delirium. Additionally, in that study, ketamine was associated with reduced norepinephrine requirement, days of benzodiazepine administration and continuous opioid infusion [29]. In our study, higher cumulative doses of ketamine increased the risk of

psychiatric symptoms in ARDS survivors up to 6 months after ICU discharge. Currently, it is

unknown why ketamine increased the risk of psychiatric symptoms in the short follow-up

outcome and not in the long-term follow-up.

For decades, midazolam as a γ-aminobutyric acid (GABA) receptor agonist has been one of

the most commonly administered sedative drugs for ICU patients [30,31]. However, a

benzodiazepine dominant sedation strategy (e.g. with midazolam) is associated with an

increased risk of developing delirium, prolonged duration of invasive mechanical ventilation

and ICU length of stay [13,32,33]. ICU delirium is common and may increase mortality risk and contribute to long-term cognitive impairment [34,35]. In our study, higher cumulative doses of midazolam did not affect the HRQoL and risk of psychiatric symptoms in ARDS survivors.

###### Strengths and limitations

The strengths of the present study are its prospective design with three follow-up time points, the large number of included patients with ARDS from hospitals across Germany and the

detailed collection of data on HRQoL, psychiatric symptoms and individual patient

characteristics. Despite our best efforts to follow up each patient, the number of drop-outs and loss of analgosedation data acquirement was rather high, which may have resulted in selection

bias limiting this study. In this post-hoc data collection only a selection of sedatives and

analgetic could be used for reasons of practicability. The instruments used for screening mental disorders do not allow making diagnoses such as major depression disorders and PTSD; thus, only symptoms or the risk of being affected by such a disorder were recorded.

# Conclusion

In summary, prolonged administration of ketamine during ICU treatment was positively associated with the risk of psychiatric symptoms. The analgesia and sedation did not influence

HRQoL of ARDS survivors up to 1 year after ICU discharge. The administration of ketamine

to ICU patients with ARDS should be with caution or refrained due to the alternative of other

sedative and analgesic drugs. Larger prospective studies are needed to analyse the influence of analgosedation and notably of ketamine during ICU treatment on the development of psychiatric symptoms in ARDS survivors.

# Declarations

### List of abbreviations

ARDS: Acute respiratory distress syndrome; BMI: Body	mass	index; HRQoL: Health‑ related quality of life; ICU: Intensive care unit; MCS‑ 12: Mental Component Summary; PCS‑ 12: Physical Component Summary; PHQ‑ 9: Patient Health Questionnaire‑ 9; PTSD: Posttraumatic stress disorders; PTSS‑ 14: Post‑ Traumatic Stress Syndrome 14‑ Questions Inventory; SAPS II: Simplified Acute Physiology Score; SF ‑ 12: Short Form‑ 12 self‑ report questionnaire; SOFA: Sequential organ failure assessment

### Ethics approval and consent to participate

The study was approved by the Ethics Committee of the University of Regensburg (file number 13-101-0262) and where necessary by Ethics Committees of the participating study centres. Written informed consent was obtained from caregivers/legal guardians and additionally from patients who had survived ICU.

### Consent for publication

Not applicable

### Availability of data and materials

The study data cannot be shared publicly because the written consent obtained from the study participants did not include a public availability of their data. Data are available from the two principal investigators (PIs) Christian Apfelbacher and Thomas Bein (E-mail: [Christian.Apfelbacher@med.ovgu.de](mailto:Christian.Apfelbacher@med.ovgu.de) and/or [Thomas.Bein@ukr.de](mailto:Thomas.Bein@ukr.de)) on request.

### Conflict of interest

CK: I have read the journal's policy and the authors of this manuscript have the following competing interests: personal fees from Maquet, personal fees from Xenios, personal fees from Bayer, non‑ financial support from Speaker of the German register of ICUs, grants from the German Ministry of Research and Education, during the conduct of the study. All other authors (SB, FZ, FDS, SuB, MB, CA, and TB) declare no conflict of interest.

### Funding

The study was funded by the German Ministry of Education and Research (Bundesministerium für Bildung und Forschung, funding number 01GY1340,

<https://www.gesundheitsforschung-bmbf.de/de/versorgungsstudien-3401.php>,	principal investigators CA and TB). The funders had no role in study design, data collection and analysis, decision to publish, or preparation of the manuscript.

### Author Contributions

**Conceptualization:** Sebastian Blecha, Magdalena Rohr, Frank Dodoo-Schittko, Susanne Brandstetter, Christian Karagiannidis, Christian Apfelbacher, Thomas Bein

**Data curation:** Sebastian Blecha, Magdalena Rohr, Susanne Brandstetter, Christian Karagiannidis,

**Formal analysis:** Sebastian Blecha, Florian Zeman, Magdalena Rohr

**Investigation:** Sebastian Blecha, Magdalena Rohr, Christian Karagiannidis

**Methodology:** Sebastian Blecha, Florian Zeman, Magdalena Rohr, Frank Dodoo-Schittko, Susanne Brandstetter, Christian Karagiannidis, Christian Apfelbacher, Thomas Bein

**Project administration:** Christian Apfelbacher, Thomas Bein

**Resources:** Sebastian Blecha, Magdalena Rohr, Christian Karagiannidis, Christian Apfelbacher, Thomas Bein

**Software:** Florian Zeman, Magdalena Rohr

**Supervision:** Susanne Brandstetter, Frank Dodoo-Schittko, Christian Karagiannidis, Christian Apfelbacher, Thomas Bein

**Validation:** Sebastian Blecha, Florian Zeman, Magdalena Rohr, Susanne Brandstetter

**Visualization:** Sebastian Blecha, Florian Zeman

**Writing – original draft:** Sebastian Blecha

**Writing – review & editing:** Florian Zeman, Magdalena Rohr, Frank Dodoo-Schittko, Susanne Brandstetter, Christian Karagiannidis, Christian Apfelbacher, Thomas Bein

# Acknowledgement

We are indebted to the highly committed intensivists and study assistants across Germany who had recruited patients for the DACAPO study:

Aachen, Aachen University Hospital RWTH Aachen, Department of Anaesthesiology (PD Dr. Johannes Bickenbach, Dr. Thorben Beeker, PD Dr. Tobias Schürholz, Jessica Pezechk);

Amberg, Klinikum Amberg, Department for Anaesthesiology (Dr. Jens Schloer); Augsburg, Klinikum Augsburg (Dr. Ulrich Jaschinski, Ilse Kummer); Bamberg, Sozialstiftung Bamberg Hospital, Department for Anaesthesiology (Dr. Oliver Kuckein); Berlin, Charité - University Medicine Berlin, Department of Anaesthesiology and Intensive Care Medicine (PD Dr. Steffen Weber-Carstens, Dr. Anton Goldmann, Dr. Stefan Angermair, Krista Stoycheva); Berlin, HELIOS Klinikum Berlin-Buch, Department of Intensive Care Medicine (Prof. Dr. Jörg Brederlau, Nadja Rieckehr, Gabriele Schreiber, Henriette Haennicke); Bielefeld, Ev. Krankenhaus Bielefeld. Department of Anaesthesiology, Intensive Care Medicine, Emergency Medicine and Pain Therapy (Dr. Friedhelm Bach, Dr. Immo Gummelt, Dr. Silke Haas, Catharina Middeke, Dr. Ina Vedder, Marion Klaproth); Bochum, Ruhr University Bochum, Department of Anaesthesiology (Prof. Dr. Michael Adamzik, Dr. Jan Karlik, Dr. Stefan Martini, Luisa Robitzky); Bonn, University Hospital Bonn, Department of Anaesthesiology and Intensive Care Medicine (Prof. Dr. Christian Putensen, Dr. Thomas Muders, Ute Lohmer); Bremen, Klinikum Bremen-Mitte, Department of Anaesthesiology (Prof. Dr. Rolf Dembinski); Deggendorf, Medical Centre, Department of Anaesthesiology and Intensive Care Medicine (Dr. Petra Schäffner, Dr. Petra Wulff-Werner); Dortmund, Klinikum Dortmund, Department of Critical Care Medicine (Elke Landsiedel-Mechenbier, Daniela Nickoleit- Bitzenberger, Ann-Kathrin Silber); Dresden, University Hospital Dresden Carl Gustav Carus, Department of Anaesthesiology and Intensive Care Medicine (Prof. Dr. Maximilian Ragaller, Prof. Dr. Marcello Gama de Abreu, Alin Ulbricht, Linda Reisbach); Frankfurt am Main, University Hospital Frankfurt, Department of Anaesthesiology, Intensive Care Medicine and Pain Therapy (Prof. Dr. Kai Zacharowski, Prof. Dr. Patrick Meybohm, Simone Lindau, Haitham Mutlak); Freiburg, University Medical Center Freiburg, Department of Anaesthesiology and Critical Care Medicine Freiburg (Prof. Dr. Alexander Hötzel, Dr. Johannes Kalbhenn); Freising, Klinikum Freising, Department of Anaesthesiology (Dr. Christoph Metz, Dr. Stefan Haschka); Göppingen, Klinik am Eichert, ALB FILS Kliniken, Department of Anaesthesiology and Intensive Care (Dr. Stefan Rauch); Göttingen, University Medical Center, Department of Anaesthesiology, Emergency and Intensive Care Medicine (Prof. Dr. Michael Quintel, Dr. Lars-Olav Harnisch, Dr. Sophie Baumann, Andrea Kernchen); Greifswald, University Medicine Greifswald, Department of Internal Medicine B (Dr. Sigrun Friesecke, Sebastian Maletzki); Hamburg, University Hospital Hamburg-Eppendorf, Department of Intensive Care Medicine, Centre of Anaesthesiology and Intensive Care Medicine (Prof. Dr. Stefan Kluge, Dr. Olaf Boenisch, Dr. Daniel Frings, Birgit Füllekrug, Dr. Nils Jahn, Dr. Knut Kampe, Grit Ringeis, Brigitte Singer, Dr. Robin Wüstenberg); Hannover,

Hannover Medical School, Department of Anaesthesiology and Intensive Care Medicine (Dr. Jörg Ahrens, Dr. Heiner Ruschulte, Dr. Andre Gerdes, Dr. Matthias Groß); Hannover, Hannover Medical School, Department of Respiratory Medicine (Dr. Olaf Wiesner, Aleksandra Bayat-Graw); Heidelberg, University of Heidelberg, Department of Anaesthesiology (Dr. Thorsten Brenner, Dr. Felix Schmitt, Anna Lipinski) ; Herford, Klinikum Herford, Clinic for Anaesthesiology, Surgical Intensive Care Medicine, Emergency Care Medicine, Pain Management (Prof. Dr. Dietrich Henzler, Dr. Klaas Eickmeyer, Dr. Juliane Krebs, Iris Rodenberg); Homburg, Homburg University Medical Centre, Department of Anaesthesiology, Intensive Care and Pain Medicine (Dr. Heinrich Groesdonk, Kathrin Meiers, Karen Salm, Prof. Dr. Thomas Volk); Ibbenbüren, Ibbenbüren General Hospital, Division of Thoracic Surgery and Lung Support (Prof. Dr. Stefan Fischer, Basam Redwan); Immenstadt, Kempten-Oberallgaeu Hospitals, Clinic for Pneumology, Thoracic Oncology, Sleep- and Respiratory Critical Care (Dr. Martin Schmölz, Dr. Kathrin Schumann-Stoiber, Simone Eberl); Ingolstadt, Klinikum Ingolstadt, Department of Anaesthesiology and Critical Care Medicine (Prof. Dr. Gunther Lenz, Thomas von Wernitz-Keibel, Monika Zackel); Jena, Jena University Hospital, Department of Anaesthesiology and Intensive Care Therapy (Dr. Frank Bloos, Dr. Petra Bloos, Anke Braune, Anja Haucke, Almut Noack, Steffi Kolanos, Heike Kuhnsch, Karina Knuhr-Kohlberg); Kassel, Klinikum Kassel, Department of Anaesthesiology (PD Dr. Markus Gehling); Kempten, Klinikum Kempten-Oberallgäu gGmbH, Department for Anaesthesia and Operative Intensive Care (Prof. Dr. Mathias Haller, Dr. Anne Sturm, Dr. Jannik Rossenbach); Kiel, University Medical Center Schleswig- Holstein, Campus Kiel, Department of Anaesthesiology and Intensive Care Medicine (Dr. Dirk Schädler, Stefanie D'Aria); Köln, Cologne-Merheim Hospital, Department of Pneumology and Critical Care Medicine (Prof. Dr. Christian Karagiannidis, Dr. Stephan Straßmann, Prof. Dr. Wolfram Windisch); Köln, University Hospital of Cologne, Department of Anaesthesiology and Intensive Care Medicine (Prof. Dr. Thorsten Annecke, PD Dr. Holger Herff); Langen, Asklepios Kliniken Langen-Seligenstadt, Department of Anaesthesiology and Intensive Care Medicine (Dr. Michael Schütz); Leipzig, University of Leipzig, Department of Anaesthesiology and Intensive Care Medicine (PD Dr. Sven Bercker, Hannah Reising, Mandy Dathe, Christian Schlegel); Ludwigsburg, Klinikum Ludwigsburg, Academic Teaching Hospital, University of Heidelberg, Department of Anaesthesiology (Katrin Lichy); Ludwigshafen, Klinikum Ludwigshafen, Department of Anaesthesiology and Intensive Care Medicine (Prof. Dr. Wolfgang Zink, Dr. Jana Kötteritzsch); Mainz, University Medical Centre Mainz, Department of Anaesthesiology (Dr. Marc Bodenstein, Susanne Mauff, Peter

Straub); Magdeburg, Magdeburg University Medical Centre, Department of Anaesthesiology and Intensive Care Medicine (Dr. Christof Strang, Florian Prätsch, Prof. Dr. Thomas Hachenberg); Mannheim, University Medical Centre Mannheim, Department of Anaesthesiology and Surgical Intensive Care Medicine (Dr. Thomas Kirschning, Dr. Thomas Friedrich, Dr. Dennis Mangold); Marburg, University Hospital, Department of Anaesthesiology (Dr. Christian Arndt, Tilo Koch); Mönchengladbach, Kliniken Maria-Hilf GmbH, Department of Cardiology (Dr. Hendrik Haake, Katrin Offermanns); München, Bogenhausen Hospital, Department of Anaesthesiology (Prof. Dr. Patrick Friederich, Dr. Florian Bingold); München, Klinikum Großhadern, Department of Anaesthesiology (Dr. Michael Irlbeck, Prof. Dr. Bernhard Zwissler); München, Klinikum Neuperlach, Städtisches Klinikum München GmbH, Department of Anaesthesiology, Critical Care and Pain Medicine (PD Dr. Ines Kaufmann); München, Klinikum rechts der Isar, Department for Anaesthesiology of the Technical University of Munich (Dr. Ralph Bogdanski, Dr. Barbara Kapfer, Dr. Markus Heim, PD Dr. Günther Edenharter); Münster, University Hospital Münster, Department for Anaesthesiology, Intensive Care Medicine and Pain Therapy, (Prof. Dr. Björn Ellger, Daniela Bause); Neumarkt, Kliniken des Landkreises Neumarkt i.d.OPf, Department for Anaesthesiology and Intensive Care Medicine (Dr. Götz Gerresheim); Nürnberg, General Hospital Nuremberg, Paracelsus Medical University, Department of Emergency Medicine and Intensive Care (Dr. Dorothea Muschner, Prof. Dr. Michael Christ, Arnim Geise); Osnabrück, Marienhospital Osnabrück, Department of Anaesthesiology (PD Dr. Martin Beiderlinden, Dr. Thorsten Heuter); Passau, Klinikum Passau, Department for Anaesthesiology (Dr. Alexander Wipfel); Regensburg, Caritas Krankenhaus St. Josef, Department for Anaesthesiology (Dr. Werner Kargl, Dr. Marion Harth, Dr. Christian Englmeier); Regensburg, Regensburg University Hospital, Department of Anaesthesiology, Operative Intensive Care (Prof. Dr. Thomas Bein, Dr. Sebastian Blecha, Dr. Kathrin Thomann-Hackner, Marius Zeder); Stuttgart, Katharinenhospital, Department of Anaesthesiology (Dr. Markus Stephan); Traunstein, Klinikum Traunstein, Department of Anaesthesiology (Dr. Martin Glaser); Tübingen, Tübingen University Hospital, Eberhard- Karls University Tübingen, Department of Anaesthesiology and Intensive Care Medicine (PD Dr. Helene Häberle); Ulm, Ulm University, Department of Anaesthesiology (Prof. Dr. Hendrik Bracht, Christian Heer, Theresa Mast); Würzburg, University of Würzburg, Department of Anaesthesia and Critical Care (PD Dr. Markus Kredel, PD Dr. Ralf Müllenbach).

Furthermore, we are grateful to previous members of the Regensburg DACAPO study team (medical documentation: Phillip Sebök, study physician: Kathrin Thomann-Hackner), to the members of the Advisory Board of the DACAPO-Study (Prof. Dr. Julika Loss, Prof. Dr. Bernhard Graf, Prof. Dr. Michael Leitzmann, Prof. Dr. Michael Pfeifer, Regensburg, Germany), to our assisting students (Simon Bein, Vreni Brunnthaler, Carina Forster, Stefanie Hertling, Sophie Höhne, Carolin Schimmele, Elisa Valletta), and we are grateful to Monika Schoell for the linguistic revision of the manuscript.

# References

1. Matthay MA, Ware LB, Zimmerman GA. The acute respiratory distress syndrome. J Clin Invest. 2012; 122: 2731–2740. doi: 10.1172/JCI60331.
2. Karagiannidis C, Mostert C, Hentschker C, Voshaar T, Malzahn J, Schillinger G, et al. Case characteristics, resource use, and outcomes of 10 021 patients with COVID-19 admitted to 920 German hospitals. An observational study. The Lancet Respiratory Medicine. 2020; 8: 853–862. doi: 10.1016/S2213-2600(20)30316-7.
3. Botta M, Tsonas AM, Pillay J, Boers LS, Algera AG, Bos LDJ, et al. Ventilation management and clinical outcomes in invasively ventilated patients with COVID-19 (PRoVENT-COVID). A national, multicentre, observational cohort study. The Lancet Respiratory Medicine. 2021; 9: 139–148. doi: 10.1016/S2213-2600(20)30459-8.
4. Nelson BJ, Weinert CR, Bury CL, Marinelli WA, Gross CR. Intensive care unit drug use and subsequent quality of life in acute lung injury patients. Crit Care Med. 2000; 28: 3626–3630. doi: 10.1097/00003246-200011000-00013.
5. Tanaka LMS, Azevedo LCP, Park M, Schettino G, Nassar AP, Rea-Neto A, et al. Early sedation and clinical outcomes of mechanically ventilated patients: a prospective multicenter cohort study. Crit Care. 2014; 18: R156. doi: 10.1186/cc13995.
6. Bienvenu OJ, Friedman LA, Colantuoni E, Dinglas VD, Sepulveda KA, Mendez-Tellez P, et al. Psychiatric symptoms after acute respiratory distress syndrome: a 5-year longitudinal study. Intensive Care Med. 2018; 44: 38–47. doi: 10.1007/s00134-017- 5009-4.
7. Bein T, Weber-Carstens S, Apfelbacher C. Long-term outcome after the acute respiratory distress syndrome: different from general critical illness. Curr Opin Crit Care. 2018; 24: 35–40. doi: 10.1097/MCC.0000000000000476.
8. Latronico N, Peli E, Calza S, Rodella F, Novelli MP, Cella A, et al. Physical, cognitive and mental health outcomes in 1-year survivors of COVID-19-associated ARDS. Thorax. 2021. doi: 10.1136/thoraxjnl-2021-218064.
9. Aranda J, Oriol I, Martín M, Feria L, Vázquez N, Rhyman N, et al. Long-term impact of COVID-19 associated acute respiratory distress syndrome. J Infect. 2021.

doi: 10.1016/j.jinf.2021.08.018.

10. Brandstetter S, Dodoo-Schittko F, Blecha S, Sebök P, Thomann-Hackner K, Quintel M, et al. Influence of quality of care and individual patient characteristics on quality of life and return to work in survivors of the acute respiratory distress syndrome. Protocol for a prospective, observational, multi-centre patient cohort study (DACAPO). BMC Health Serv Res. 2015; 15: 563. doi: 10.1186/s12913-015-1232-2.
11. Dodoo-Schittko F, Brandstetter S, Brandl M, Blecha S, Quintel M, Weber-Carstens S, et al. German-wide prospective DACAPO cohort of survivors of the acute respiratory distress syndrome (ARDS). A cohort profile. BMJ Open. 2018; 8: e019342.

doi: 10.1136/bmjopen-2017-019342.

12. Dodoo-Schittko F, Brandstetter S, Brandl M, Blecha S, Quintel M, Weber-Carstens S, et al. Characteristics and provision of care of patients with the acute respiratory distress syndrome. Descriptive findings from the DACAPO cohort baseline and comparison with international findings. J Thorac Dis. 2017; 9: 818–830. doi: 10.21037/jtd.2017.03.120.
13. Ranieri VM, Rubenfeld GD, Thompson BT, Ferguson ND, Caldwell E, Fan E, et al. Acute respiratory distress syndrome. The Berlin Definition. JAMA. 2012; 307: 2526– 2533. doi: 10.1001/jama.2012.5669.
14. Blecha S, Brandl M, Zeman F, Dodoo-Schittko F, Brandstetter S, Karagiannidis C, et al. Tracheostomy in patients with acute respiratory distress syndrome is not related to quality of life, symptoms of psychiatric disorders or return-to-work. The prospective DACAPO cohort study. Ann. Intensive Care. 2020; 10: 2731. doi: 10.1186/s13613-020- 00671-x.
15. Kroenke K, Spitzer RL, Williams JB. The PHQ-9. Validity of a brief depression severity measure. J Gen Intern Med. 2001; 16: 606–613. doi: 10.1046/j.1525- 1497.2001.016009606.x.
16. Parker AM, Sricharoenchai T, Raparla S, Schneck KW, Bienvenu OJ, Needham DM. Posttraumatic stress disorder in critical illness survivors. A metaanalysis. Crit Care Med. 2015; 43: 1121–1129. doi: 10.1097/CCM.0000000000000882.
17. Bullinger M. German translation and psychometric testing of the SF-36 Health Survey. Preliminary results from the IQOLA project. Social Science & Medicine. 1995; 41: 1359–1366. doi: 10.1016/0277-9536(95)00115-n.
18. Stieglitz R-D. Bullinger, M. & Kirchberger, I. (1998). SF-36. Fragebogen zum Gesundheitszustand. Göttingen. Hogrefe. Preis DM 298,-. Zeitschrift für Klinische Psychologie und Psychotherapie. 1999; 28: 143–145. doi: 10.1026//0084-5345.28.2.143.
19. Piriyapatsom A, Bittner EA, Hines J, Schmidt UH. Sedation and paralysis. Respir Care. 2013; 58: 1024–1037. doi: 10.4187/respcare.02232.
20. Strøm T, Martinussen T, Toft P. A protocol of no sedation for critically ill patients receiving mechanical ventilation. A randomised trial. The Lancet. 2010; 375: 475–480. doi: 10.1016/S0140-6736(09)62072-9.
21. Ren X, Ai Y, Zhang L, Zhao C, Li L, Ma X. Sedation and analgesia requirements during venovenous extracorporeal membrane oxygenation in acute respiratory distress syndrome patients. Perfusion. 2021: 2676591211052160. doi: 10.1177/02676591211052160.
22. Stevenson JE, Colantuoni E, Bienvenu OJ, Sricharoenchai T, Wozniak A, Shanholtz C, et al. General anxiety symptoms after acute lung injury: predictors and correlates. J Psychosom Res. 2013; 75: 287–293. doi: 10.1016/j.jpsychores.2013.06.002.
23. Herridge MS, Tansey CM, Matté A, Tomlinson G, Diaz-Granados N, Cooper A, et al. Functional disability 5 years after acute respiratory distress syndrome. N Engl J Med. 2011; 364: 1293–1304. doi: 10.1056/NEJMoa1011802.
24. Mion G, Villevieille T. Ketamine pharmacology. An update (pharmacodynamics and molecular aspects, recent findings). CNS Neurosci Ther. 2013; 19: 370–380.

doi: 10.1111/cns.12099.

25. Fond G, Loundou A, Rabu C, Macgregor A, Lançon C, Brittner M, et al. Ketamine administration in depressive disorders. A systematic review and meta-analysis. Psychopharmacology (Berl). 2014; 231: 3663–3676. doi: 10.1007/s00213-014-3664-5.
26. Serafini G, Howland RH, Rovedi F, Girardi P, Amore M. The role of ketamine in treatment-resistant depression. A systematic review. Curr Neuropharmacol. 2014; 12: 444–461. doi: 10.2174/1570159X12666140619204251.
27. Köhler S, Betzler F. Ketamin - Eine neue Option in der Behandlung der therapieresistenten Depression. Fortschr Neurol Psychiatr. 2015; 83: 91–97. doi: 10.1055/s-0034-1398967.
28. Wu TT, Ko S, Kooken R, van den Boogaard M, Devlin JW. Exploring Ketamine Analgosedation Use and Its Effect on Incident Delirium in Critically Ill Adults. Crit Care Explor. 2021; 3: e0544. doi: 10.1097/CCE.0000000000000544.
29. Jaeger M, Attridge RL, Neff LA, Gutierrez GC. Safety and Effectiveness of Sedation With Adjunctive Ketamine Versus Nonketamine Sedation in the Medical Intensive Care Unit. J Pharm Pract. 2021; 34: 850–856. doi: 10.1177/0897190020925932.
30. Martin J, Franck M, Fischer M, Spies C. Sedation and analgesia in German intensive care units. How is it done in reality? Results of a patient-based survey of analgesia and sedation. Intensive Care Med. 2006; 32: 1137–1142. doi: 10.1007/s00134-006-0214-6.
31. Patel RP, Gambrell M, Speroff T, Scott TA, Pun BT, Okahashi J, et al. Delirium and sedation in the intensive care unit. Survey of behaviors and attitudes of 1384 healthcare professionals. Crit Care Med. 2009; 37: 825–832.

doi: 10.1097/CCM.0b013e31819b8608.

32. Casault C, Soo A, Lee CH, Couillard P, Niven D, Stelfox T, et al. Sedation strategy and ICU delirium. A multicentre, population-based propensity score-matched cohort study. BMJ Open. 2021; 11: e045087. doi: 10.1136/bmjopen-2020-045087.
33. Fraser GL, Devlin JW, Worby CP, Alhazzani W, Barr J, Dasta JF, et al. Benzodiazepine versus nonbenzodiazepine-based sedation for mechanically ventilated, critically ill adults. A systematic review and meta-analysis of randomized trials. Crit Care Med. 2013; 41: S30-8. doi: 10.1097/CCM.0b013e3182a16898.
34. Ely EW, Shintani A, Truman B, Speroff T, Gordon SM, Harrell FE, et al. Delirium as a predictor of mortality in mechanically ventilated patients in the intensive care unit. JAMA. 2004; 291: 1753–1762. doi: 10.1001/jama.291.14.1753.
35. Zaal IJ, Devlin JW, Peelen LM, Slooter AJC. A systematic review of risk factors for delirium in the ICU. Crit Care Med. 2015; 43: 40–47.

doi: 10.1097/CCM.0000000000000625.

# Supporting information

**S1 Table:** Multiple linear regression models of the influence of cumulative analgosedation (without ketamine and midazolam) on psychiatric symptoms in ARDS survivors 3, 6 and 12 months after ICU discharge**.**

S1 Table legend: *p <0.05; **per 1000 mg; ^#^further adjusted for age, sex, SAPS, days on ICU; B: regression coefficient, 95%-CI: 95% confidence interval, PHQ-9: Patient Health Questionnaire-9; PTSS-14: Post-Traumatic Stress Syndrome 14-Questions Inventory; ^a^symptoms of psychiatric disorders (depression and PTSD) were diagnosed according to the results of patient self-reported questionnaires

# Figures legends

**Fig. 1:** Flow chart of patients with ARDS throughout the study

**Fig. 2:** Relationship between cumulative dose of ketamine and psychiatric symptoms in ARDS survivors 3, 6 and 12 months after ICU discharge

**Fig. 3:** Relationship between cumulative dose of midazolam and psychiatric symptoms in ARDS survivors 3, 6 and 12 months after ICU discharge

# Tables legends

**Tab. 1:** Patient characteristics of ARDS survivors (n=134)

**Tab. 2:** Total cumulative dose and duration of sedation and analgesia in ARDS survivors (n=134)

**Tab. 3:** HRQoL and symptoms of psychiatric disorders in ARDS survivors after 3, 6 and 12 months (n=84)

**Tab. 4:** Multiple linear regression models of the influence of cumulative ketamine doses on psychiatric symptoms in ARDS survivors 3, 6 and 12 months after ICU discharge

Response to Reviewers

Response letter

Dear Mrs. Linda L. Maerz,

Thank you for the opportunity to revise our manuscript entitled: “Association of analgosedation with psychiatric symptoms and health-related quality of life in ARDS survivors: Post hoc analyses of the DACAPO study”. We are very grateful for the positive review, helpful suggestions, and comments of the reviewers and you to improve our paper. We have thoroughly modified the manuscript accordingly. Detailed responses to the reviewers’ comments are listed below.

Journal Requirements:

When submitting your revision, we need you to address these additional requirements.

1. Comment (C): Please ensure that your manuscript meets PLOS ONE's style requirements, including those for file naming.

Answer (A): We modified the whole manuscript including the title page and file naming.

2. C: We noticed you have some minor occurrence of overlapping text with a previous publication.

A: We rephrase parts of the methods section and cited our previous publication (page 4 to 5).

3. C: In your Data Availability statement, you have not specified where the minimal data set underlying the results described in your manuscript can be found. PLOS defines a study's minimal data set as the underlying data used to reach the conclusions drawn in the manuscript and any additional data required to replicate the reported study findings in their entirety. All PLOS journals require that the minimal data set be made fully available. For more information about our data policy, please see <http://journals.plos.org/plosone/s/data-availability>. Important: If there are ethical or legal restrictions to sharing your data publicly, please explain these restrictions in detail. Please see our guidelines for more information on what we consider unacceptable restrictions to publicly sharing data: <http://journals.plos.org/plosone/s/data-> availability#loc-unacceptable-data-access-restrictions. Note that it is not acceptable for the authors to be the sole named individuals responsible for ensuring data access.

A: The conceptual design of the informed consent did not allow sharing of the results of the self-report questionnaires. We described it in more detail in the Declarations section (page 15).

“The study data cannot be shared publicly because the written consent obtained from the study participants did not include a public availability of their data. Data are available from the two principal investigators (PIs) Christian Apfelbacher and Thomas Bein (E-mail: [Christian.Apfelbacher@med.ovgu.de](mailto:Christian.Apfelbacher@med.ovgu.de) and/or [Thomas.Bein@ukr.de](mailto:Thomas.Bein@ukr.de)) on request.”

4. C: Address all items indicated by tracked changes in the attached Word document (PONE-D-22-09940 [ed])

A: All questions which were addressed could hopefully be adequately answered. Please look at the revised manuscript with tracked changes.

Reviewer #1:

1. Comment (C): Perhaps the main concern is the interaction of different sedatives/analgesics used in the management of ARDS patients included in this evaluation. It is common to switch between therapies or administer concomitant sedatives/analgesics to achieve the appropriate level of sedation. It was not clear if these interactions were accounted for. The study results indicate that ketamine was associated with an increased risk of psychiatric symptoms. However, it's not clear if any administration of ketamine was associated with these findings or if a certain cohort of ketamine patients (those who received other sedatives or analgesics) had an increased risk of psychiatric symptoms. For example, patients who received ketamine and midazolam might have had an increased risk of psychiatric symptoms versus patients who received ketamine and propofol. Furthermore, it would be helpful if authors report in detail the breakdown of sedatives and analgesics (number of patients who received one sedative, two sedatives, and three sedatives, etc.)

A: Thank you for your critical note. Our data do not allow us to say, which drugs were switched against each other or which were stopped first. We added the multiple drug application to the multiple linear regression analyses but found no influence on the existing results. As stated below we hope we could explain our interesting findings in more detail (page 8 and 10).

“In the following, the number of sedative and analgesic agents that patients received during ICU treatment are listed (based on Table 2): 12 patients (8.9%) got a sixfold, 28 patients (20.9%) fivefold, 38 patients (28.4%) fourfold, 34 patients (25.4%) a threefold,

19 patients (14.2%) twofold and only 3 patients (2.2%) single analgosedation.”

“Thirty-two of 39 patients (82.1%) with ketamine application and all patients with esketamine additionally received midazolam.”

“The number of drugs for analgosedation showed no influence on the psychiatric outcome in the multivariable linear regression analysis.”

2. C: The other concern in the design of this evaluation is variation in practice. The study utilized the DACAPO database. Different institutions have various sedatives and analgesic dosing regimens, titration protocols goals, and ARDS management guidelines. It would be helpful to account in the multivariable analysis for the type of institutions and variation in practice.

A: We were very thankful to the reviewer for this important note. We addressed this fact in more detail in the methods section (page 5 and 6).

“The type of analgesia and sedation, duration and cumulative doses of drugs were extracted and transmitted from the patient data management system of nine recruiting study centres (8 University hospitals and one hospital for basic and standard care).”

3. C: It was not clear from the study's design whether all sedatives and analgesics extracted from the database were accounted for. According to Table 2, none of the patients received commonly used sedatives and analgesics such as dexmedetomidine, fentanyl, morphine, or lorazepam infusion. For clarity, please comment in the study design section whether these variables were included in the DACAPO database or not.

A: In this post-hoc data retrieval of all DACAPO study centres, only a selection of sedatives and analgetic (including dexmedetomidine) could use for reasons of practicability. We describe this fact in the limitations section (page 14).

4. C: In the results section, report the number of subjects with positive outcomes findings. Specifically, patients with PHQ-9-score ≥5 and PTSS-14-score ≥45.

A: Thank you once again. We explained it more accurately in Table 3.

5. C: The multivariable linear regression results focused on ketamine and midazolam. It would be helpful to report the results for other analgosedatives as a supplementary appendix table.

A: We added this supporting information as Supplement material (Table S1).

6. C: The discussion section of the manuscript might benefit from discussing why the higher prevalence of psychiatric symptoms was detected at 3- and 6-month and not at the 12-month time point.

A: We discussed this fact in our DACAPO study team, but currently it is not clear, why higher cumulative doses increase the risk of psychiatric symptoms in ARDS survivors up to 6 months after ICU discharge. We clarify this fact in the discussion section.

“Currently, it is unknown why ketamine increased the risk of psychiatric symptoms in the short follow-up outcome and not after long-term follow-up.”

7. C: The conclusion recommends that ketamine be used for a short duration. The findings of this evaluation don't support this recommendation. The study didn't investigate whether the increased incidence of psychiatric symptoms was related to the longer versus shorter duration of use. It might not be appropriate to recommend a shorter duration of ketamine use over a longer duration of use. I would suggest rephrasing the conclusion and recommendations based on the findings of this study.

A: We understand your view and modified the conclusion more accurately.

“The administration of ketamine to ICU patients with ARDS should be with caution.” (Abstract) and “The administration of ketamine to ICU patients with ARDS should be with caution or refrained due to the alternative of other sedative and analgesic drugs.” (Conclusions)

8. C: Minor	edits	(Page	4: Study	design: change	DACOPO	to	DACAPO; Page 4: Sample: change 1.225 patients to 1,225 patients (comma versus period); Page 5: First paragraph. Clarify the number of patients who died after discharge from the ICU. Figure 1 indicates N=161 versus N=158; Page 5: Consider changing "The kind of	analgesia	and	sedation"	to	"The	type	of	analgesia	and	sedation" Page 5: "For the screening tools, the cut-off values for being at risk of depression were defined as PHQ-9 (≥5) and for PTSD as PTSS-14 [≥45])." The parenthesis in this phrase is confusing.

A: Thank you for the helpful review. We adapted it according to the revision of the manuscript.

9. C: Please update.Page 7: Please reword "Sedation was often increased with clonidine (73.1%) and midazolam (59.0%)". It's not clear if this refers to an increase in depth/level of sedation or dose of sedatives.

A: We explained it correctly.

"Sedation was often deepened with clonidine (73.1%) and/or midazolam (59.0%)."

10. C: Table 3: Please clarify n=84 in the table legend. Nowhere in the manuscript was a discussion around that number of patients. Please include a detailed explanation of how this number was achieved in the manuscript text.

A: We explained this fact more detailed in the results section (page 7): „From 134 patients, where medication was documented during ICU therapy, the values of SF-12, PHQ-9, and PTSS-14 of 84 ARDS survivors could be compared.“

Reviewer #2:

Given that 262 respondents were excluded due to unknown doses and duration of sedation and analgesia application, can the author comment on impact on their conclusions? In other words, would documented dosages of sedatives during their ICU treatment in these respondents changed the conclusion that prolonged ketamine use lowered health quality of life scores?

Answer: Thank you for your helpful comment. In connection with reviewer 1, we have described this fact in more detail in the limitations section and in the conclusions. We know about the number of drop-outs and loss of analgosedation data acquirement, which may have resulted in selection bias and limited this study. Due to technical reason (e.g. lack of electronic patient data management system) a registration of analgosedation data was not possible of each DACAPO study centre. But all received analgosedation data of the recruiting study centres were checked twice for plausibility and accuracy from our DACAPO study group.

“Despite our best efforts to follow up each patient, the number of drop-outs and loss of analgosedation data acquirement was rather high, which may have resulted in selection bias limiting this study. In this post-hoc data collection only a selection of sedatives and analgetic could be used for reasons of practicability.”

“The administration of ketamine to ICU patients with ARDS should be with caution.” (Abstract)

Reviewer #3:

The data was basically exploratory as the investigators used data from the prospective observational nation- wide ARDS study across Germany (DACAPO) to investigate the influence of sedation and analgesia on HRQoL and the risk of psychiatric symptoms in ARDS survivors 3, 6 and 12 months after their discharge from the intensive care unit (ICU). The analysis was routinely performed using univariate and multivariable procedures as noted in the statistical analysis section of the paper. The tables and figures are well formatted and described. The conclusions appear to follow reasonably from the analyses performed. There are some deficiencies noted:

1. Comment (C): Although retrospective and descriptive, the sample size was 134 subjects and some statistical justification should be given for the adequacy of the sample.

A: All patients of the prospective DACAPO study with data on analgosedation were used for this supplementary analysis. Thus, no more than n=134 patients were available. Nevertheless, this sample is sufficient for the first exploratory analyses regarding our additional questions. As a rule of thumb, a minimum of 10-12 cases is needed per variable for a linear regression model. In our multiple linear regression model with eight independent variables, we needed at least 80 cases for the first exploratory analyses. (page 6)

2. In Table 3 missing data is noted especially at six months which is about 26% of the sample in some cases assuming a denominator of 84 at most. The authors need to give this some attention and explain how the missing data issue is addressed, if at all. There is no mention in the statistical analysis section of how this is handled.

A: Missing data were not imputed in this analysis. A patient with missing data was omitted from the respective analysis. We added a statement to the statistical analysis section (page 5 to 6)

We hope that we have been able to answer the questions adequately and thank you for the critical evaluation of our paper.

Kind regards, Sebastian Blecha Anaesthesiologist

Department of Anaesthesiology

University Medical Centre Regensburg Franz-Josef-Strauss-Allee 11

93053 Regensburg Germany

Phone:	+49 941 944 7801

Facsimile:	+49 941 944-7802 Email:	[Sebastian.Blecha@ukr.de](mailto:Sebastian.Blecha@ukr.de)
