# Supplementary material for: Association of analgosedation with psychiatric symptoms and health-related quality of life in ARDS survivors: Post hoc analyses of the DACAPO study
Source: PLoS One. 2022 Oct 21;17(10):e0275743. doi: 10.1371/journal.pone.0275743 (PMC9586389; doi:10.1371/journal.pone.0275743)
Supplement: Attachment — Submitted filename: Response to Reviewers 120922.docx [file pone.0275743.s005.docx]

**Response letter**

Dear Mrs. Linda L. Maerz,

Thank you for the further opportunity to revise our manuscript entitled: “Association of analgosedation with psychiatric symptoms and health-related quality of life in ARDS survivors: Post hoc analyses of the DACAPO study”. We are very grateful for the positive review, the helpful suggestions, and the comments of the reviewers and academic editor to improve our manuscript. We have thoroughly modified the manuscript accordingly. Detailed responses to the reviewers’ comments are listed below.

**Journal Requirements:**

**Comment:** Please review your reference list to ensure that it is complete and correct. If you have cited papers that have been retracted, please include the rationale for doing so in the manuscript text, or remove these references and replace them with relevant current references. Any changes to the reference list should be mentioned in the rebuttal letter that accompanies your revised manuscript. If you need to cite a retracted article, indicate the article’s retracted status in the References list and also include a citation and full reference for the retraction notice.

**Answer:** We reviewed the reference list of our last uploaded version. To the best of our knowledge, all journal abbreviations were corrected. As stated below, on the basis of the editor's helpful recommendations the following references were added to the manuscript compared to the original version:

Reference 14: Blecha S, Brandl M, Zeman F, Dodoo-Schittko F, Brandstetter S, Karagiannidis C, et al. Tracheostomy in patients with acute respiratory distress syndrome is not related to quality of life, symptoms of psychiatric disorders or return-to-work. The prospective DACAPO cohort study. Ann. Intensive Care. 2020; 10: 2731. doi: 10.1186/s13613-020-00671-x

Reference 33: Fraser GL, Devlin JW, Worby CP, Alhazzani W, Barr J, Dasta JF, et al. Benzodiazepine versus nonbenzodiazepine-based sedation for mechanically ventilated, critically ill adults. A systematic review and meta-analysis of randomized trials. Crit Care Med. 2013; 41: S30-8. doi: 10.1097/CCM.0b013e3182a16898.

Reference 34: Pun BT, Badenes R, La Heras Calle G, Orun OM, Chen W, Raman R, et al. Prevalence and risk factors for delirium in critically ill patients with COVID-19 (COVID-D). A multicentre cohort study. Lancet Respir Med. 2021; 9: 239–250. doi: 10.1016/S2213-2600(20)30552-X.

Additionally, the reference changes were highlighted now in the "Revised Manuscript with Track Changes".

**Academic editor:**

**Answer:** Thank you for the helpful comments. All questions were answered accurately as possible and are highlighted in the "Revised Manuscript with Track Changes".

**Reviewer #1:**

The authors updated the manuscript based on the feedback provided. Thank you for allowing me to review the revised manuscript. The authors addressed all the major and minor edit requests. I have two minor feedback points:

1. **Comment:** Please update the header with clear labels for easier understanding of the table. Columns 2 and 3 of Table 3 don’t have labels. Also, consider moving “mean (+/-SD)” and “n (%)” to these column labels.

**Answer:** Thanks for this important point. Now, we explain the headings in table 3 more accurately for better readability and understanding.

2. **Comment:** Consider an alternative word to “refrained” in the conclusion section, “The administration of ketamine to ICU patients with ARDS should be with caution or refrained due to the alternative of other sedative and analgesic drugs”. Consider: The administration of ketamine to ICU patients with ARDS should be with caution or avoided as alternative sedative and analgesic drugs are available.

**Answer:** Thank you for this note. We rephrased this sentence in the conclusion section.

**Reviewer #2:**

This is an important research about a commonly used sedative in the ICU that would influence patient care and outcomes. Larger prospective study about ketamine would be beneficial to the community for the future.

**Answer:** Thank you for your positive feedback, intellectual support, and interest in our manuscript.

We hope that we have been able to answer the questions adequately and thank you for the critical evaluation of our manuscript.

Kind regards,

Sebastian Blecha

Anaesthesiologist

Department of Anaesthesiology

University Medical Centre Regensburg

Franz-Josef-Strauss-Allee 11

93053 Regensburg

Germany

Phone:	+49 941 944 7801

Facsimile:	+49 941 944-7802

Email:	Sebastian.Blecha@ukr.de
